# Genetic diversity and population structure analysis of Philippine native pigs highlight five priority populations for conservation

**DOI:** 10.1002/ece3.10618

**Published:** 2023-10-31

**Authors:** Joy B. Banayo, Kathlyn Louise V. Manese, Kaito O. Furusho, Agapita J. Salces, Takahiro Yamagata

**Affiliations:** ^1^ Animal Genetics and Breeding, Department of Animal Science, Graduate School of Bioagricultural Sciences Nagoya University Nagoya Japan; ^2^ Animal Breeding Division, Institute of Animal Science, College of Agriculture and Food Science University of the Philippines Los Baños Los Baños Laguna Philippines

**Keywords:** genetic diversity, indigenous pig, livestock conservation, microsatellite, population structure

## Abstract

The Philippine native pig (PhNP) is a unique genetic resource composed of multiple domesticated *Sus scrofa* lineages and interspecific hybrids. No prior study has determined the population structure and genetic diversity of PhNPs on multiple islands and provinces, which is essential for establishing conservation priorities. In this study, we explore the population structure and genetic diversity of various PhNP populations in Luzon and the Visayas, Philippines, to identify conservation priorities. We analyzed seven PhNP populations (*n* = 20–27 samples each; Benguet [B], Kalinga [K], Nueva Vizcaya [N], Isabela [I], Quezon [Q], Marinduque [M], and Samar [S]) and four transboundary breeds present in the Philippines (*n* = 9–11 samples each; Duroc, Large White, Landrace, and Berkshire). The pigs were compared against a panel of 20 microsatellite markers recommended by the ISAG–FAO. We tested for population structure at the island, region, and province levels. Strong genetic differentiation between native and transboundary breeds was confirmed by Bayesian clustering (*k* = 2) and Nei's *D*_*A*_ genetic distance (100% bootstrap support for the PhNP cluster). PhNP exhibited high heterozygosity (*Ho*: 0.737), a high allele count (*Na*: 7.771), and a low inbreeding coefficient (*F*is: −0.040–0.125). Bayesian clustering supported genetic differentiation at the island (*k* = 2; North Luzon and South Luzon‐Visayas cluster), region (*k* = 3), and population (*k* = 8) levels. The pairwise *F*'st between PhNP populations ranged from 0.084 (N and I) to 0.397 (Q and K), confirming that some PhNP populations exhibited sufficient genetic distance to be considered separate populations. This study shows that native pigs from B, K, I, Q, M, and S are unique genetic units for conservation. Furthermore, the small effective population sizes of B, I, Q, M, and S (*Ne*: 3.9, 19.1, 14.2, 44.7, and 22.5, respectively) necessitate immediate conservation actions, such as incentivizing PhNP farming.

## Graphical abstract

The Philippine native pig is a unique genetic resource consisting of multiple domesticated pig lineages and interspecies hybrids. Using microsatellite analysis, we demonstrate the population structure at the provincial level that supports six locally recognized populations. Low effective population sizes require immediate conservation actions.

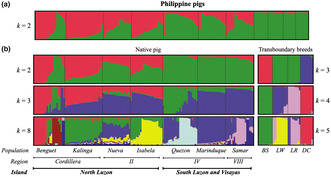

## 1 INTRODUCTION

Effective agricultural biodiversity conservation is important for sustainable food systems (Sponenberg, 2020). Biodiversity has three levels, as outlined by Hvilsom et al. (2022): ecosystem diversity, species diversity, and genetic diversity, defined as the “amount and distribution of genetic variation within species or populations.” To safeguard genetic diversity, monitoring using molecular tools is fundamental for the evaluation of population trajectories to support the implementation of preventive actions, such as the conservation of critical and distinct populations (Bruford et al., 2017; Hvilsom et al., 2022; Notter, 1999). In recognition of the global decline in biodiversity, the Food and Agriculture Organization (FAO, 2011) justified the inclusion of understudied populations, such as the Philippine native pig (PhNP), as an important component of biodiversity. Various studies have suggested that in these otherwise low‐productivity stocks, favorable alleles can exist, which may contribute to future selection responses (Notter, 1999). The prioritization of breeds in terms of conservation can be based on various genetic diversity measures, such as allelic diversity, heterozygosity, and population structure (Fernandez & Bennewitz, 2018). For instance, population structure analysis of the indigenous pigs of Vietnam showed that among the initial 15 breeds, there are only nine distinct populations worthy of conservation (Van Ba et al., 2020). Furthermore, genetic diversity and structure analysis of the Iberoamerican livestock breeds justified the recognition of the Creoles breed of livestock and therefore subsequent conservation programs (Gama et al., 2020). On the other hand, population structure studies can inform management decisions to control the damage caused by feral and wild pigs (Delgado‐Acevedo et al., 2021; Hampton et al., 2004). In this study, we explored the genetic diversity and population structure of the PhNP to inform conservation.

The PhNP is locally regarded as an indigenous species adapted to local conditions (FAO, 2011; Santiago et al., 2016). It is an Asian‐type *Sus scrofa* pig belonging to the East, Southeast, Pacific, and Cordillera clades, distinct from the wild pigs of the Philippines (i.e., *S. philippensis* and *S. celebensis*; Banayo et al., 2023; Layos, Geromo, et al., 2022; Layos, Godinez, et al., 2022). PhNPs are traditionally associated with the indigenous communities who use them in rituals and to prepare ethnic foods, such as *etag* and *kinuday* (Alabado, 2017; Garambas et al., 2022; Lapena & Acabado, 2017; Ma, 2010; Maddul et al., 2015; Molintas, 2017; Voss, 1987). Academic interest in PhNP has attracted strong government support for its conservation and genetic characterization (Senate Bill 821, Philippine Native Animal Development Act of 2019). Local initiatives to promote PhNP have focused on supporting PhNP farming, especially for the *lechon* market*. Lechon* (roasted whole pig) is an important cultural delicacy in the Philippines, and PhNP is an ideal ingredient due to its superior meat quality (Santiago et al., 2016; Sarian, 2019). However, despite the demand for PhNP *lechon*, PhNPs are still primarily reared in an extensive production system characterized by traditional management, small herd sizes, low productivity, and low income (Falculan, 2021; Maddul, 1991; Manilay et al., 2021; Muth et al., 2020; Quintua et al., 2019). The actual proportion of PhNP is not known and is pending a national inventory, but PhNP is known to be a minority breed in the Philippines with a proportion ranging from 1.4% to 4% (Armenia et al., 2016; Barnes et al., 2020; Bondoc & Ramos, 1995), except in the Cordillera region of North Luzon, where a proportion of up to 74% has been reported (Maddul, 1991). Similar to other Asian pigs (Charoensook et al., 2019; Michalk et al., 2019; Zhang et al., 2016, 2018), the low productivity of the PhNP is traditionally bolstered by crossbreeding with transboundary breeds (Arboleda et al., 2003). Thus, the genetic integrity of the PhNP may have been compromised.

**FIGURE 1:**
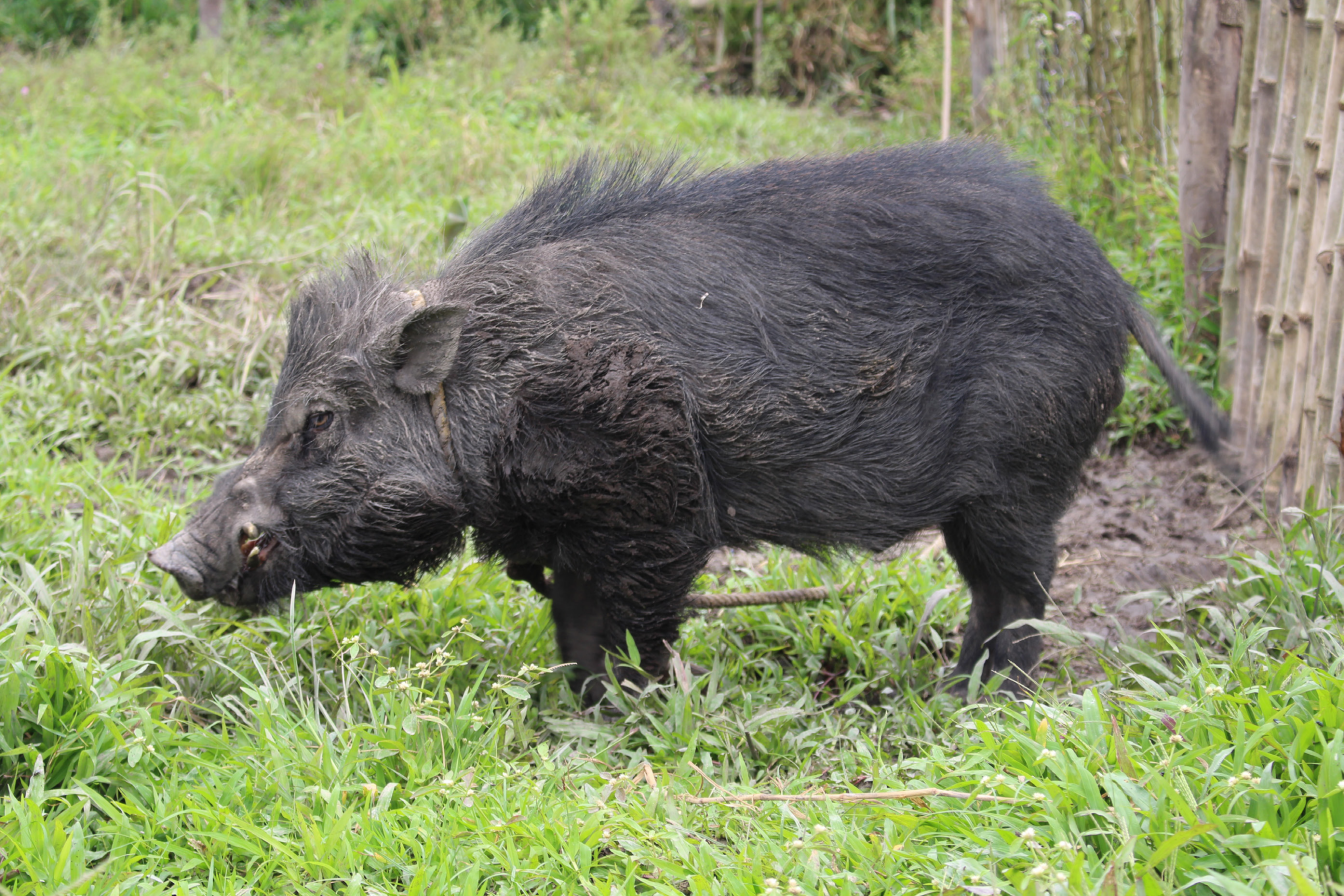
A not so rare male Philippine native pig from the Kalinga population. This male pig is widely used as a sire in mating systems. Local researcher Ms. Sharmaine D. Codiam claims it to be an interspecific hybrid between a Philippine native and a wild pig from Tanudan, Kalinga. Photographed in 2017 by J. B. Banayo at the University Farm, Kalinga State University, Bulanao, Tabuk City, Kalinga, Philippines.

Recent phylogenetic analyses indicated that PhNP may have at least four origins of domestication throughout Asia, that is Southeast Asia (SEA), East Asia (EA), the Cordillera region/Lanyu Island, and the Pacific (Banayo et al., 2023; Dichoso et al., 2022; Larson et al., 2005; Layos, Geromo, et al., 2022; Layos, Godinez, et al., 2022; Tanaka et al., 2008; Wu et al., 2007). These multiple ancestries suggest high genetic variation among PhNPs on different islands. Furthermore, it revealed that the Cordillera Clade (which includes the Benguet [B] population) has ancestry distant from the general Asian clade (Banayo et al., 2023; Basilio Jr. et al., 2022), similar to the findings in the related Lanyu pig of Taiwan (Li et al., 2017; Wu et al., 2007). Moreover, PhNP interbreeds with endemic wild pigs of the Philippines, such as *Sus philippensis*, *S. cebifrons*, *S. ahoenobarbus*, *S. olivieri*, and *S*. sp. (from Sulu), further complicating PhNP genetics (Figure 1) (Groves, 1997; Heaney et al., 2020; Ingicco et al., 2017; Lucchini et al., 2005). Population structure analysis using microsatellite markers showed that the PhNP was distinct from Duroc (DC) and Yorkshire pigs (Oh et al., 2014). Recently, Logronio et al. (2022) also showed that neighboring PhNP populations on southern Luzon in Quezon (Q) and Marinduque (M) were moderately differentiated despite their proximity. We therefore hypothesized that the population structure of the PhNP is unique and may provide clues for sustainable conservation practices and maximum utilization of these native pigs. Aside from the Q and M populations mentioned, PhNP subpopulations, despite being named after their geographic origins, have no genetic basis for distinction.

This study aimed to gain a deeper understanding of the genetic diversity and population structure of the PhNP by microsatellite analysis and comparison of their molecular variance at the island, administrative region, and population levels, with the goal of informing genetic improvement and conservation programs. We analyzed seven populations from Luzon and the Visayas in the Philippines and identified B, Q, I, and S as priority populations for conservation. We recommend new conservation actions to complement existing ones, such as providing incentives to PhNP farmers for performing agroecological services and to further encourage the farming of PhNPs. This study shows the strong stratification of populations in an island archipelago, providing a genetic basis for the high level of mammalian endemism in the Philippines.

## 2 MATERIALS AND METHODS

### 2.1 Samples and sampling sites

**FIGURE 2:**
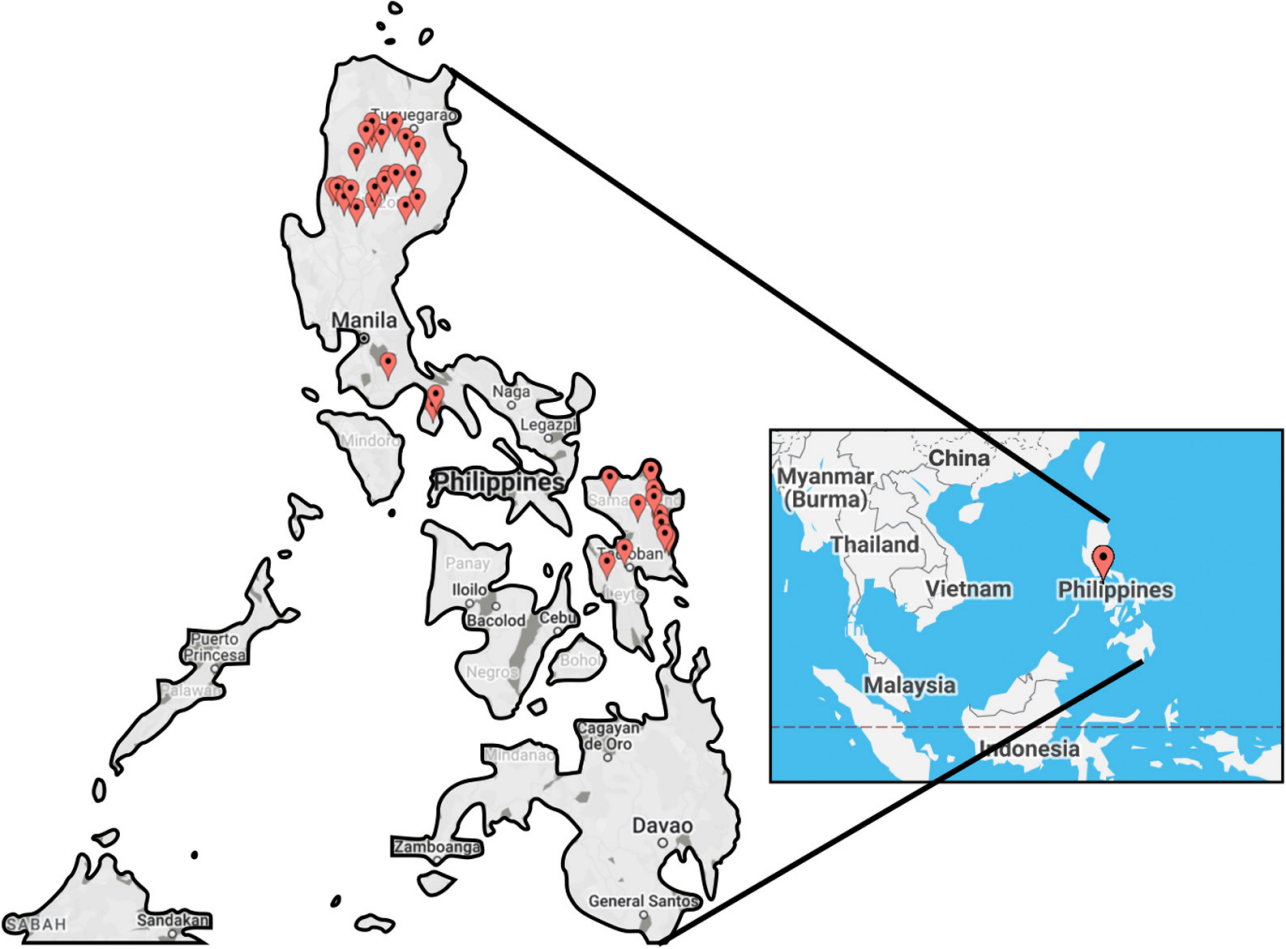
Sites at which native pigs were sampled in the Philippines. Red pins indicate municipalities in the following provinces: Benguet (Bokod, Sablan, Itogon, Tublay, and La Trinidad), Mountain Province (Bauko), Kalinga (Tabuk City, Lubuagan, Tinglayan, Pasil, and Tanudan), Isabela (Cordon, Gamu, Echague, and Mallig), Nueva Vizcaya (Bayombong, Bagabag, Dupax del Norte, Sta. Fe and Diadi), Quirino (Maddela and Nagtipunan), Quezon (Tiaong), Marinduque (Torrijos and Sta. Cruz), Northern Samar (Gamay and Lope De Vega), Eastern Samar (Llorente, Borongan City, Balangkayan, Dolores, Can‐avid, and San‐Julian), Samar (Paranas), and Leyte (Ormoc City and Alang‐alang). Map created by BatchGeo (www.batchgeo.com) with data from Google ©2022.

The Philippines is a Southeast Asian country located at latitude 11°48′10.80″ N and longitude 122°33′46.80″ E (OSM, 2021). There are at least 7641 islands composed primarily of volcanic rock and coral (Borlaza, 2022; Larena et al., 2021; NAMRIA, 2017, 2018). The islands are subdivided into three major island groups: Luzon in the north, Visayas in the middle, and Mindanao in the south (UN, 2022). The Cordillera (Central) Mountain Range, the central mountain chain of Luzon, is the most prominent range, with an average elevation of approximately 1800 m, while most of the central plain of Luzon is only approximately 30 m above sea level (Borlaza, 2022). The populations considered in this study were purposively collected from provinces in Luzon and Visayas with PhNP farms and collaborating local researchers (Figure 2). Native pig raising in the Philippines is mostly a family tradition; average engagement was reported at 20 years (Maddul, 1991; Monleon, 2005), and each family maintains an average herd size of five animals (Banayo, unpublished) that are raised in a low‐input system (Ayomen & Kingan, 2019; Maddul, 1991; Manipol et al., 2014; Monleon, 2005; Quintua et al., 2019). For farming purposes, PhNPs have limited movement, where the purchase of animals is restricted to the neighborhood (Maddul, 1991; Quintua et al., 2019); however, adult pigs raised for *lechon* can reach major cities via middlemen, such as Metro Manila, the capital of the Philippines, for processing (Lesaca, 2016). Where free‐range practices are implemented, PhNPs are known to escape to the wild. Notably, raising PhNP mostly serves as a means of savings and for cultural traditions rather than as a major income source (Ayomen & Kingan, 2019; Maddul, 1991).

Native pigs were sampled through hair or ear notches in collaboration with local researchers between 2014 and 2021, and the majority were collected before the outbreak of African swine fever (ASF) disease occurred in the Philippines. At least 40 animals were sampled based on FAO (2011) recommendations, but we genotyped only at least 20 animals per population. In choosing animals, we sampled one animal per family or, if free‐range, a maximum of five animals per village and represented at least three municipalities per province. A total of 5 administrative regions (CAR, II, IVA, IVB, and VIII), 14 provinces, and 36 municipalities were represented (Figure 2, Table A1). In rare cases where the farm was large, up to five unrelated animals were included, and the relations of animals were based on testimonials of the owners. Population names were assigned based on the province where the majority of samples were collected. These populations were as follows: Benguet (B), *n* = 22 samples from Benguet and Mountain Province; Kalinga (K), *n* = 27 samples; Nueva Vizcaya (N), *n* = 20 samples from Nueva Vizcaya and Quirino; Isabela (I), *n* = 23 samples; Quezon (Q), *n* = 25 samples; Marinduque (M), *n* = 20 samples; and Samar (S), *n* = 20 samples from Eastern, Northern Samar, Samar (formerly Western Samar) and Leyte. Samples from international transboundary breeds, pigs that can be found in many countries, were collected from accredited breeding farms in the Philippines as follows: Large White (LW), *n* = 11 samples; Landrace (LR), *n* = 9 samples; and Duroc (DC), *n* = 9 samples. These three breeds are the major pig breeds farmed in the Philippines and were used as references to verify admixture by crossbreeding, as well as provide a reference for assessing PhNP from an international perspective (FAO, 2011). Additionally, Berkshire (BS), *n* = 10 samples, were collected from the government facility, National Swine and Poultry Research and Development Center, Bureau of Animal Industry (NSPRDC‐BAI). BS has been intensively used in the past for the upgrading of PhNP (Arboleda et al., 2003).

### 2.2 DNA extraction and PCR amplification

Twenty‐one microsatellite markers for swine (Table 1) recommended by the FAO/International Society of Animal Genetics (ISAG) were used in 3‐plex multiplex groups, preferably, or single‐plex (FAO, 2011). DNA was extracted from ear notch samples (20 mg) or hair follicles (five hairs) using a GF‐1 Tissue DNA Extraction Kit (Vivantis Technologies Sdn Bhd, Malaysia) following the manufacturer's protocol. The PCR solution contained 20 ng of DNA, 1× SuperPlex™ Premix (Takara Bio, USA), and 0.05–0.10 μM each of the primers in either multiplex or single‐plex reactions, for a total volume of 25 μL. Forward primers were 5′ labeled with either 6‐FAM, HEX or TAMRA (Applied Biosystems, USA). The thermal cycle profile was 95°C for 2 min, 35 cycles of [95°C for 10 s, primer‐specific annealing temperature for 20 s (Table 1), and 72°C for 15 s], and a final extension of 72°C for 2 min. Fragment analysis (FA) was performed on a 3730XL DNA analyzer (Applied Biosystems) using the GeneScan™ 400HD ROX™ internal lane standard (Applied Biosystems). We also sequenced the PCR product per primer to confirm dinucleotide repeat motifs (Table 1). Samples were manually scored using Peak Scanner v1.0 (Applied Biosystems).

**TABLE 1:** The microsatellite markers used for the genetic characterization of Philippine pigs.

Primer name	Forward sequence (5′–3′)	Reverse sequence (5′–3′)	Chr^a^	T_A_ ^b^	MG^c^	5′ dye	Repeat motif^d^
S0005	TCCTTCCCTCCTGGTAACTA	GCACTTCCTGATTCTGGGTA	5	60	1	TAMRA	GT
S0155	TGTTCTCTGTTTCTCCTCTGTTTG	AAAGTGGAAAGAGTCAATGGCTAT	1		1	FAM	CA
S0026	AACCTTCCCTTCCCAATCAC	CACAGACTGCTTTTTACTCC	16		1	HEX	–
S0355	TCTGGCTCCTACACTCCTTCTTGATG	TTGGGTGGGTGCTGAAAAATAGGA	15	60	2	TAMRA	CA
Sw830	AAGTACCATGGAGAGGGAAATG	ACATGGTTCCAAAGACCTGTG	10		2	FAM	GT
Sw2410	ATTTGCCCCCAAGGTATTTC	CAGGGTGTGGAGGGTAGAAG	8		2	HEX	GT
Swr1941	AGAAAGCAATTTGATTTGCATAATC	ACAAGGACCTACTGTATAGCACAGG	13	60	3	TAMRA	GT
Sw632	TGGGTTGAAAGATTTCCCAA	GGAGTCAGTACTTTGGCTTGA	7		3	FAM	CA
Sw24	CTTTGGGTGGAGTGTGTGC=	ATCCAAATGCTGCAAGCG	17		3	HEX	GT
S0228	GGCATAGGCTGGCAGCAACA	AGCCCACCTCATCTTATCTACACT	6	60	4	FAM	CA
S0218^e^	GTGTAGGCTGGCGGTTGT	CCCTGAAACCTAAAGCAAAG	X		4	HEX	CA
Sw936	TCTGGAGCTAGCATAAGTGCC	GTGCAAGTACACATGCAGGG	15		4	TAMRA	GT
S0097	GACCTATCTAATGTCATTATAGT	TTCCTCCTAGAGTTGACAAACTT	4	58	5	FAM	CA
Sw857	TGAGAGGTCAGTTACAGAAGACC	GATCCTCCTCCAAATCCCAT	14		5	TAMRA	GT
Sw122	TTGTCTTTTTATTTTGCTTTTGG	CAAAAAAGGCAAAAGATTGACA	6		5	HEX	GT
Sw2406	AATGTCACCTTTAAGACGTGGG	AATGCGAAACTCCTGAATTAGC	6	58	6	TAMRA	GT
IGF1	GCTTGGATGGACCATGTTG	CATATTTTTCTGCATAACTTGAACCT	5		6	FAM	CA
Sw240	AGAAATTAGTGCCTCAAATTGG	AAACCATTAAGTCCCTAGCAAA	2		6	HEX	–
S0090	CCAAGACTGCCTTGTAGGTGAATA	GCTATCAAGTATTGTACCATTAGG	12	58	7	TAMRA	CA/TA
S0226	GCACTTTTAACTTTCATGATACTCC	GGTTAAACTTTTNCCCCAATACA	2		7	FAM	TG
Sw72	ATCAGAACAGTGCGCCGT	TTTGAAAATGGGGTGTTTCC	3		7	HEX	GT

*Note*: Sequences and chromosome locations were adapted from the Food and Agriculture Organization (FAO, 2011).

^a^ Chromosome location.

^b^ Annealing temperature.

^c^ Multiplex group.

^d^ Based on partial sequencing.

^e^ Sex marker only — this pertains to column Repeat Motif, wherein the motif was not successfully sequenced.

### 2.3 Statistical analysis

A global test of composite linkage disequilibrium (LD) between alleles at two loci (regardless of chromosome location) was conducted using default parameters in GENEPOP (Rousset, 2008) and LINKDOS (Garnier‐Géré & Dillmann, 1992). All *p*‐values were adjusted by controlling the false discovery rate (FDR) by using the formula: rank* m/*k*, where *k* is the position of a *p*‐value in the sorted vector and *m* is the number of independent tests (Benjamini & Hochberg, 1995; Jafari & Ansari‐Pour, 2019).

For each microsatellite marker and for each population, the number of different alleles (*Na*), the effective number of alleles (*Ea*), Shannon's information index (*I*), observed (*Ho*) and expected (*He*) heterozygosity, the fixation index (*F*), the F‐statistics (*F*is, *F*it, *F*st), and the effective number of migrants *(Nm)* were estimated with GENALEX v6.5 (Peakall & Smouse, 2006, 2012) from all samples (*n* = 196). The polymorphic information content (*PIC*) was computed from all samples by POWERMARKER (Liu & Muse, 2005). Deviation from Hardy–Weinberg equilibrium (HWE) was tested from all samples using two different assumptions in GENEPOP v4.7.5 (Raymond & Rousset, 1995; Rousset, 2008): (i) the assumption of heterozygote excess to test for genotyping error, and (ii) the assumption of heterozygote deficiency to test for population structure. The effective population size (*Ne*) was computed in NEESTIMATOR v2.1 (Do et al., 2014) using its LD method with the lowest allele frequency set to 0.01. Regional *F*‐statistics were computed by analysis of molecular variance (AMOVA) following the methods of Meirmans (2006) and Weir and Cockerham (1984) using the codom‐allelic setting (assuming an infinite allele model [IAM]) and the codom‐microsatellite setting (assuming a stepwise mutation model [SMM]), with at least 999 permutations as implemented in GENALEX v6.5. In the AMOVA, we evaluated the variance in genetic diversity attributable to five regional distributions ([i] native versus transboundary breeds [*n* = 2], [ii] North Luzon [NL] vs. South Luzon and Visayas [SLV; *n* = 2], [iii] administrative region [Cordillera, II, IV, and VIII; *n* = 4], [iv] high vs. low elevation [*n* = 2], and [v] native_high, native_low, and transboundary [*n* = 3]) as well as variance due to between‐population differences (*n* = 11, all populations; *n* = 7, PhNPs only) and between‐individual differences (within population), using allelic identity and allelic size. The standardized pairwise *F*'st (Weir & Cockerham, 1984) between populations (*n* = 11) was computed via AMOVA using the codom‐allelic setting, with 999 permutations as implemented in GENALEX v6.5. Dendrograms were constructed using the unweighted pair group method with arithmetic mean (UPGMA) approach, the neighbor‐joining (NJ) method and Nei's *D*_*A*_ genetic distance matrix (Nei et al., 1983; Takezaki & Nei, 2008) generated in DISPAN (Ota, 1993) with 10,000 replicates.

Bayesian clustering analysis was implemented in STRUCTURE v2.3.4 (Pritchard et al., 2000) to examine the genetic structure of pigs using an admixture ancestry model and correlated allele frequencies, with or without location input (LOCPRIOR), using all samples or PhNPs only (*n* = 157). Ten independent runs were performed at each *k* (1–16 for all samples and for PhNPs, and 1–5 for transboundary breeds). Each run consisted of 1,000,000 Markov chain Monte Carlo (MCMC) replicates after a 1,000,000 burn‐in. The resulting *f*‐files from STRUCTURE were then analyzed using STRUCTURE HARVESTER v0.6.94 (Earl & VonHoldt, 2012) as implemented in PYTHON v3 (Rossum & Drake, 2009). Using the STRUCTURE HARVESTER package, the ad hoc statistic ΔK was calculated with the Evanno method (Evanno et al., 2005) to determine the optimal number of clusters. For each value of K, K*.indfile and K*.popfile were then generated using the *–clumpp* function in preparation for use with CLUMPP. The generated fields were then used in CLUMPP (Jakobsson & Rosenberg, 2007) to generate Q‐matrices (.indivq and .popq files), and the optimal alignment of independent runs was obtained with the LargeKGreedy algorithm with 10,000 repeats. To visualize the Q‐matrices, DISTRUCT (Rosenberg, 2004) was used to generate membership probability plots. The raw data reported here are available in the Dryad database (Banayo et al., 2022).

## 3 RESULTS

To determine the genetic diversity of the PhNP, we first aimed to determine basic genetic information, such as the informativeness of the microsatellite markers, the number of alleles in each population, and the effective population sizes, to inform conservation priorities. We obtained a total of 260 alleles with lengths of 86–272 bp (Table A2). Each marker showed an average *Ea* of 3.673 and *PIC* of 0.779 (Table A2). One of the 20 markers (Sw24) deviated from HWE (Table A2). Furthermore, marker‐marker LD analysis revealed 131 out of 190 locus pairs to be in LD (Table S1). Most (77%) were found in the B population (Table S2). Further analysis showed 110 locus pairs with an allele fixed at one or both loci (Table S3). A high degree of LD is expected around loci under selection, based on the principle of genetic hitchhiking (Kirk & Freeland, 2011; Pfaffelhuber et al., 2008). However, genome‐scale analysis also showed that an impression of high LD can be expected in structured populations showing differentiated loci (fixed in some and variable in others), such as our study population (Li et al., 2019). We recognize that the main issue is that markers in LD do not meet the neutrality expectations for genetic analysis. Thus, LD analysis is necessary in studies such as microsatellite panel development (Miller et al., 2019). However, for this study, the panel of markers recommended by the ISAG‐FAO had to be used as a complete set to allow for a global comparison of animal genetic diversity (FAO, 2011). Furthermore, unlike high‐density SNP panels, the markers are dispersed, with 1–2 loci per chromosome (Table 1). Therefore, they are not physically linked on the same chromosome, which eliminates the need for marker pruning. Finally, no marker pruning was performed, and we proceeded to use 20 microsatellite markers to assess the genetic diversity of PhNP according to the FAO (2011).

Population analysis showed that the PhNP had an average of 7.771 alleles (*Na*), 4.366 effective alleles (*Ea*), and 0.279 private alleles (*Pa*) across loci (Table 2). The highest allele count among PhNPs (*Na*: 8.850) was observed in the N population, while the lowest (*Na*: 6.850) was in the B population. The observed heterozygosity (*Ho)* was 0.737. The B, K, and S populations deviated from HWE according to the heterozygote deficiency method, suggesting further population stratification. The inbreeding coefficient suggested no to moderate inbreeding among PhNPs (*F*is: −0.040 to 0.125; Table 2). Meanwhile, the transboundary breeds had a lower number of alleles (*Na*: 3.800; *Ea*: 2.460) and lower heterozygosity (*Ho*: 0.582; Table 2).

**TABLE 2:** Genetic diversity of 11 populations of pigs in the Philippines based on 20 microsatellite loci.

Population (R/P)		*n*	*Na*	*Ea*	*I*	*Pa*	*Ho*	*He*	*F*	*P*_HWE_^EXC^	*P*_HWE_^DEF^	*F*is	*Ne*
NL	B	22	6.850	3.906	1.563	0.050	0.666	0.75	0.090	2.000	.000^a^	0.113	3.9
	K	27	8.150	3.840	1.548	0.400	0.662	0.71	0.042	2.219	.011^a^	0.065	125.9
	N	20	8.850	5.032	1.810	0.400	0.795	0.81	−0.009	2.874	.459	0.015	69.9
	I	23	7.300	4.255	1.621	0.300	0.798	0.77	−0.066	.495	2.057	−0.040	19.1
SLV	Q	25	7.500	4.003	1.557	0.100	0.758	0.73	−0.053	2.515	1.061	−0.034	14.2
	M	20	7.800	4.502	1.693	0.400	0.776	0.78	−0.014	2.529	.460	0.012	44.7
	S	20	7.950	5.021	1.759	0.300	0.706	0.80	0.099	2.000	.000^a^	0.125	22.5
Mean native		22.3	7.771	4.366	1.650	0.279	0.737	0.764	0.013				
T	BS	10	2.600	1.930	0.695	0.050	0.530	0.44	−0.254	.008^a^	1.998	−0.210	19.7
	LW	10	4.600	2.923	1.179	0.000	0.631	0.64	−0.037	2.488	1.256	0.017	16.7
	LR	9	4.550	2.761	1.117	0.100	0.534	0.60	0.035	2.476	.048	0.114	133.0
	DC	9	3.450	2.224	0.877	0.150	0.633	0.53	−0.270	.000^a^	1.818	−0.222	‐
Mean transboundary		9.5	3.800	2.460	0.967	0.075	0.582	0.552	−0.131				
Mean all samples		17.6	6.327	3.673	1.402	0.205	0.681	0.687	−0.038				
SE		0.442	0.173	0.104	0.032		0.012	0.012	0.012				

Abbreviations: B, Benguet; BS, Berkshire; DC, Duroc; *Ea*, no. of effective alleles; *F*, fixation index; *F*is, inbreeding coefficient; *He*, unbiased expected heterozygosity; *Ho*, observed heterozygosity; I, Isabela; *I*, Shannon's information index; K, Kalinga; LR, Landrace; LW, Large White; M, Marinduque; *n*, sample size; *Na*, no. of alleles; *Ne*, effective population size; NL, North Luzon region; NV, Nueva Vizcaya; *P*, probability value in Hardy–Weinberg equilibrium test using heterozygote excess (HWE^EXC^), and heterozygote deficiency method (HWE^DEF^), *p*‐values were adjusted using FDR; *Pa*, number of private alleles; Q, Quezon; R/P, Region/province; S, Samar; SE, standard error; SLV, South Luzon and Visayas region; T, transboundary commercial breeds.

^a^ Significant at alpha = .05.

We further assessed the sources of diversity in the PhNP to determine whether it is attributable to the individual, the subpopulation, or to any of the five regional distributions, in particular highland versus lowland PhNPs, and tested two mutation models (SMM and IAM). The AMOVA results indicated that the variation in genetic diversity between highland and lowland PhNPs, attributable to region, population (between‐population level), and individual (within‐population level), was 18%, 5%, and 78%, respectively, when allele size was incorporated into the analysis (SMM; Table 3). Subsequently, the regional variation between highland and lowland PhNP was high (*F*st: 0.226). Furthermore, the regional difference in highland PhNPs, lowland PhNPs, and transboundary breeds was 7%. In general, lower values were obtained using IAM. Our results show that the SMM attributes larger variance to the regional differences between highland and lowland PhNPs, indicating that it is a better model than IAM when the analysis includes the highland PhNPs (B population).

**TABLE 3:** Analysis of molecular variance in Philippine native pigs and commercial transboundary breeds.

Genetic distance model	Sample	Region	Number of populations	Variance components (%)			*F*‐statistics				
				Within population	Between populations within regions	Between regions	*F*st	*F*sr	*F*rt	*F*'rt	*F*'sr
Allele Size (SMM^a^)	Native	High^c^, Low^d^	7	78	5	18	0.226	0.056	0.180		
	All	High, Low, Transboundary^e^	11	88	5	7	0.116	0.050	0.070		
Allelic Identity (IAM^b^)	Native	High, Low	7	94	5	1	0.059	0.053	0.006	0.032	0.227
	All	High, Low, Transboundary	11	88	9	3	0.121	0.095	0.029	0.143	0.349

^a^ Stepwise mutation model.

^b^ Infinite allele model.

^c^ High—native pig from a highland elevation (the B population).

^d^ Low—native pig from lowland elevation (the K, I, N, Q, M, and S populations).

^e^ Duroc, Large White, Landrace, and Berkshire.

The high between‐population variation among PhNPs was further dissected by pairwise analysis using standardized *F'st*. Between the NL and SLV populations, the pairwise *F*'st ranged from 0.145 to 0.397 (Table 4), suggesting strong genetic differentiation between populations on different islands. Within the islands, pairwise *F*'st values were moderate, *F'st* between the NL populations (B, K, N, I) ranged from 0.084 to 0.175, and those between the SLV (Q, M, S) ranged from 0.137 to 0.183. On the other hand, low *F'st* values (below 0.150) were observed between the following populations: N and I, N and M, and Q and M. Recent gene flow between these populations was supported by a high number of migrants (*Nm*: 9.290 to 10.138; Table A3). Logronio et al. (2022) also reported low genetic differentiation (*F*st: 0.018) between the Q and M populations. The results indicate that PhNP populations from different islands (NL and SLV) are more likely to have stronger genetic differentiation than those from the same island. On the other hand, pairwise *F*'st showed high differentiation between the PhNPs and transboundary breeds, with values ranging from 0.328 (Q vs. LR pigs) to 0.714 (K vs. BS pigs) (Table 4). Relatively lower gene flow between PhNP and transboundary breeds was obtained (*Nm*: 0.733–3.152).

**TABLE 4:** Genetic distances between populations based on pairwise standardized *F'st*.

Population^a^		B	K	N	I	Q	M	S	BS	LW	LR	DC
NL	B	0.000										
	K	0.163	0.000									
	N	0.153	0.164	0.000								
	I	0.175	0.150	0.084	0.000							
SLV	Q	0.346	0.397	0.174	0.279	0.000						
	M	0.326	0.379	0.145	0.243	0.137	0.000					
	S	0.312	0.317	0.152	0.248	0.183	0.156	0.000				
T	BS	0.700	0.714	0.616	0.667	0.563	0.542	0.614	0.000			
	LW	0.581	0.693	0.485	0.564	0.440	0.372	0.446	0.650	0.000		
	LR	0.626	0.639	0.432	0.538	0.328	0.351	0.455	0.654	0.410	0.000	
	DC	0.673	0.700	0.510	0.556	0.535	0.485	0.579	0.761	0.618	0.611	0.000

^a^ Abbreviations of populations are shown in the footnote of Table 2.

**FIGURE 3:**
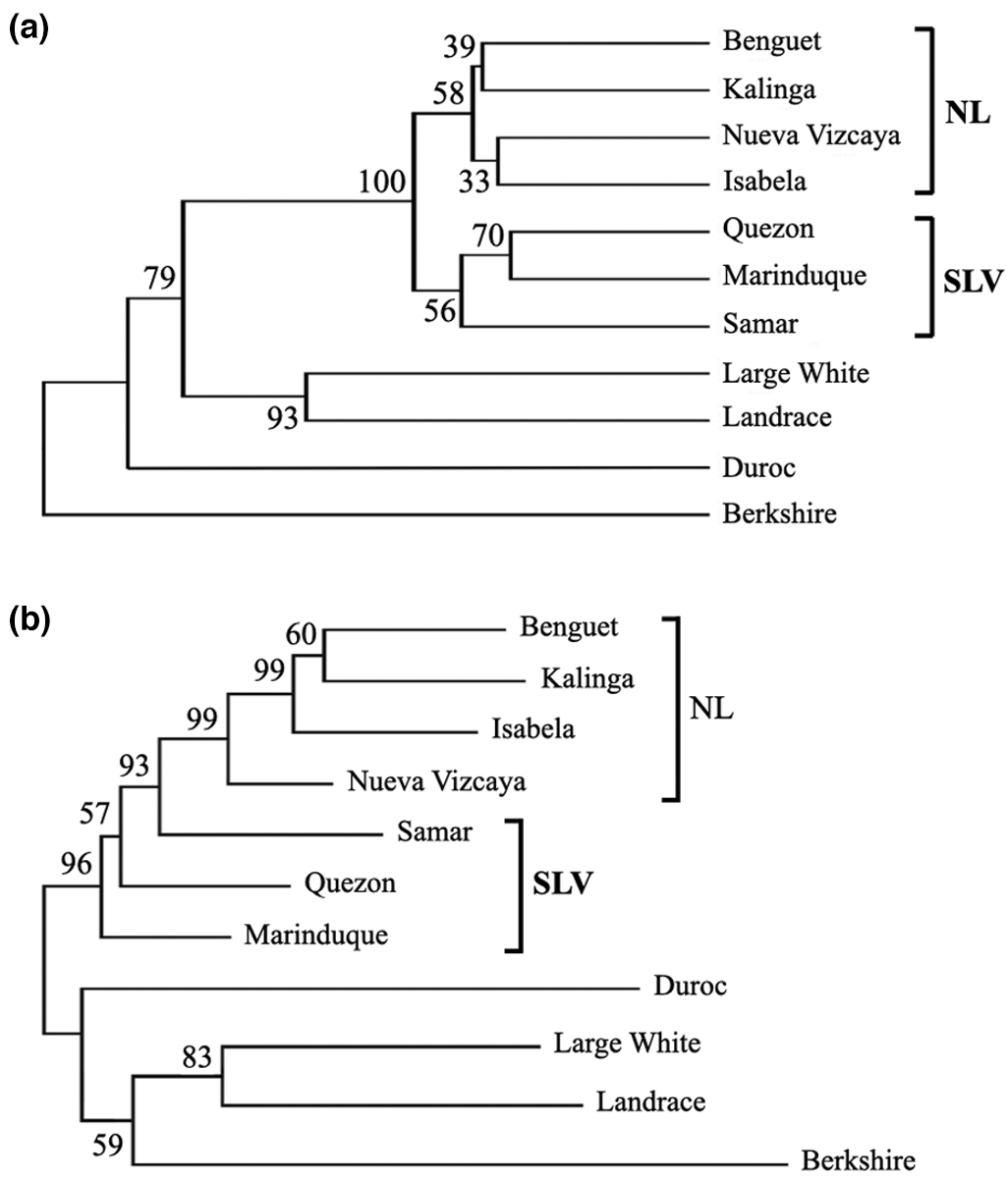
Genetic relationships among Philippine native pigs and transboundary breeds based on Nei's *D*_*A*_ distance using 20 microsatellite markers. Constructed by (a) the unweighted pair group method with arithmetic mean (UPGMA) and (b) the neighbor‐joining method. NL, North Luzon; SLV, South Luzon and Visayas; and T, transboundary breeds. Genetic distances and trees were generated by Dispan.

To further assess genetic diversity, Nei's *D*_*A*_ genetic distances were computed, and dendrograms were constructed. All PhNPs formed a distinct cluster (96%–100% bootstrap support in the UPGMA and NJ trees, respectively) from the transboundary breeds (Figure 3a, b). The NL cluster (the B, K, N, and I populations) had 58%–99% bootstrap support in the UPGMA and NJ trees, respectively. However, the cluster including the Q, M, and S populations obtained only moderate support (56%), suggesting sufficient genetic differentiation between the SL and V populations (Figure 3a). Among the SL populations, a branch containing only the Q and M populations obtained high bootstrap support (70%; Figure 3a). The pairwise *D*_*A*_ distances between the populations ranged from 0.136 to 0.507 (Table A3). This result indicates strong genetic differentiation among the NL, SL, and V populations and the occurrence of a recent gene flow between SL populations Q and M.

**FIGURE 4:**
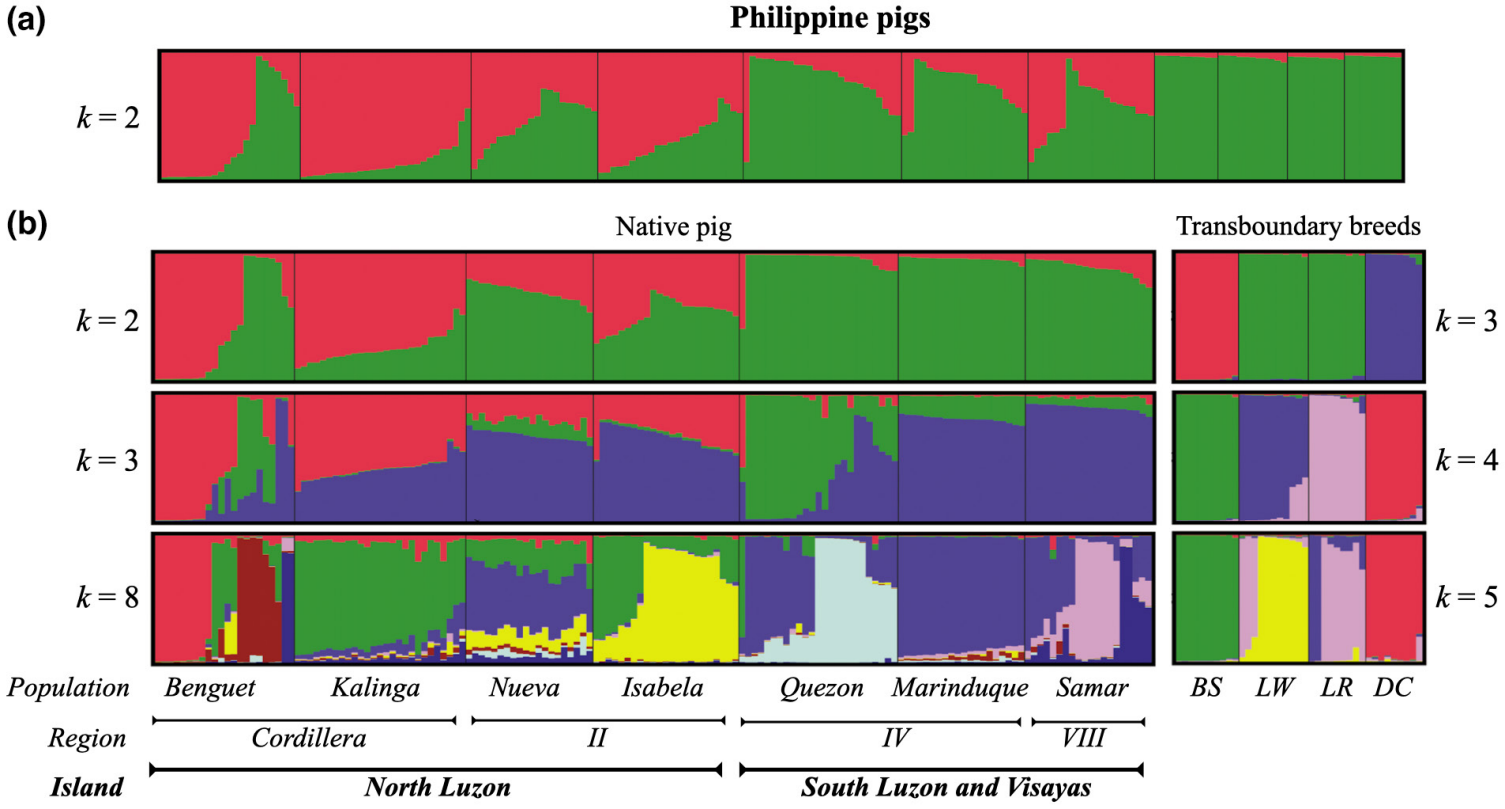
Inferred population structure of Philippine native pigs and transboundary breeds using 20 microsatellite markers clustered by the Bayesian method. Each vertical bar indicates one individual partitioned into *k* colored segments representing its estimated membership fractions in *k* clusters. (a) all pigs, (b) separate analyses of the seven native populations and four transboundary breeds. The optimal number of clusters for native was *k* = 2, 3, and 8 (with location input). Nueva refers to Nueva Vizcaya population, Region—Philippine administrative regions, BS, Berkshire; LW, Large White; LR, Landrace; and DC Duroc.

**FIGURE A1:**
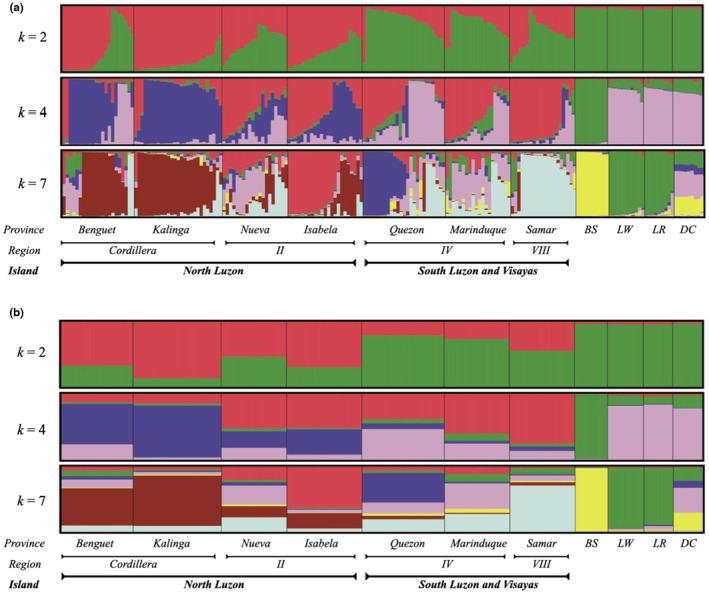
Inferred structure of combined Philippine native and transboundary breeds at the optimal number of clusters *k* = 2, 4, and 7. (a) each line represents the membership of one individual to *k* clusters, (b) average membership coefficient of each population.

**FIGURE A2:**
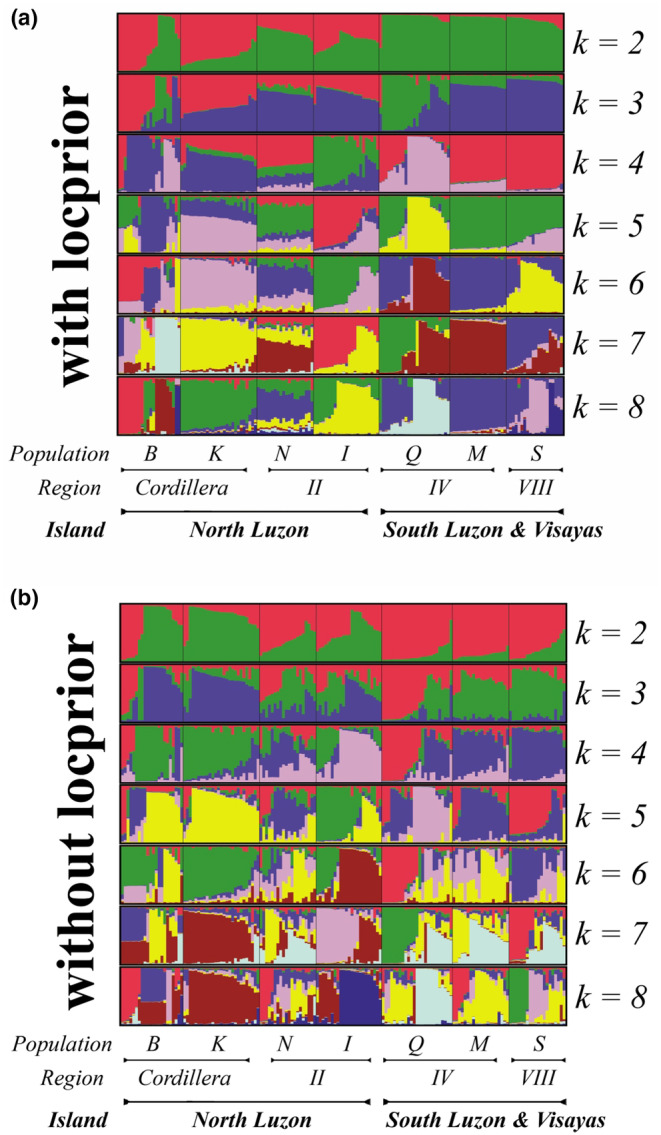
Inferred structure of Philippine native pigs at the optimal number of clusters with (a) or without (b) location information. The optimal number of clusters was (a) *k* = 2, 3, and 8; (b) *k* = 2, 3 and 4. Each line represents the membership of one individual to *k* clusters.

**FIGURE A3:**
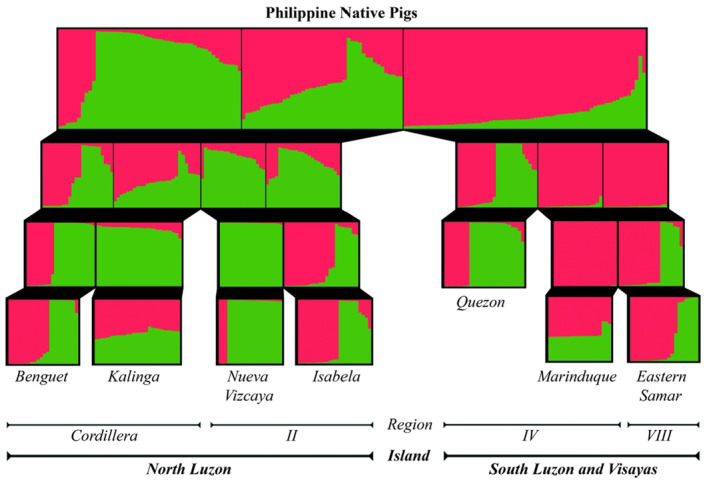
Inferred structure of Philippine native pigs at *k* = 2 hierarchical clusters without location information. Each line represents the membership of one individual to *k* clusters.

To further assess the presence of distinct PhNP populations, we used model‐based clustering analysis implemented in STRUCTURE, which assigns individuals to subpopulations based on likelihood analysis (Porras‐Hurtado et al., 2013; Pritchard et al., 2000). The results showed that the optimal number of clusters (when all samples were included) was *k* = 2, confirming the differentiation between the PhNP and transboundary breeds (Figure 4a). This number of clusters was followed by *k* = 4, and 7, in decreasing order of optimality (Figure A1a,b). On the other hand, further analysis of PhNP populations alone showed that the optimal number of clusters within PhNPs was *k* = 2, 3, 8 and *k* = 2, 3, 4, with or without location input, respectively (Figure 4 and Figure A2). The PhNP was genetically structured by island, region, and province (Figure 4). At least six populations (B, K, I, Q, M, and S) are distinct. The N population appears to be admixed with alleles from B, I, and M. Meanwhile, B, I, Q, and S were further subdivided, suggesting genetic differences between municipalities (Figure 4, Figures A2 and A3). The K and M populations appear to be genetically homogenous, suggesting that gene flow is outward from these populations.

To inform conservation priorities, we determined the effective population size *(Ne)* of each of the seven previously assigned PhNP populations. The effective population size ranged from 3.9 (the B population) to 125.9 (the K population), with the majority (except K and N) having critical *Ne* values below 50 (Table 2). The *Ne* of the M population was 44.7, which was lower than the 2010 estimate (*Ne*: 65) of Monleon et al. (2010) based on herd size. The low and declining effective population sizes in PhNPs highlight the need for immediate conservation actions to prevent further loss of genetic diversity.

## 4 DISCUSSION

This paper aimed to explore the population structure and genetic diversity of various PhNP populations in Luzon and Visayas and to identify conservation priorities. Our study showed that the PhNP exhibited high allelic richness and a population structure (Table 2) with at least six distinct populations (Figure 4). AMOVA showed that among the PhNPs, the B population was the most differentiated but suffered from low effective population size. The majority of the PhNPs (B, I, Q, M, and S populations) have a critically low *Ne* (below 50), necessitating the need for immediate conservation actions. We also show the low admixture between native and transboundary breeds, which should be maintained in favor of conservation of the distinct PhNP populations. The Philippine government supports the conservation of native animals through the Philippine Native Animal Development (PNAD) program, where the NSPRDC is the implementing arm for the ex situ conservation of native pigs (Philippine Native Animal Development Act of 2019). Currently, a limited number of PhNPs (Q and K populations) are conserved at the Tiaong Stock farm of the NSPRDC, with the goal of including all populations in the future (*personal communication*, Dr. Rene C. Santiago, Center Chief). Furthermore, the Department of Science and Technology‐Philippine Council for Agriculture, Aquatic and Natural Resources Research and Development (DOST‐PCAARRD) supports research and development projects of state universities and colleges (SUCs) that encourage PhNP farming and increased productivity and profitability (PCAARRD, 2022).

In PhNP, we identified higher allelic diversity and heterozygosity (Table 2) than the values reported in indigenous pigs of Bulgaria, Croatia, and Greece, but lower values than those reported in South Africa and the Iberian pig (Fabuel et al., 2004; Gvozdanović et al., 2019; Ishikawa et al., 2021; Michailidou et al., 2014; Swart et al., 2010). Our results show the importance of PhNP as a repository of alleles, and provide clues about how its fitness has been improved despite production constraints.

At least six populations were distinct (B, K, I, Q, M, and S) and showed further subdivision below provincial boundaries (Figure 4). Genetic substructure could be possible at the municipality level especially for B, I, Q, and S. The homogenous pattern of K and M populations could be due to cultural preference for K native pigs within K, and the local ordinance in M that prohibits outside pigs from entering the island (*personal communication*, Dr. Arnolfo M. Monleon, Professor in M). Our previous analysis of the mitochondrial DNA (mtDNA) D‐loop region revealed three ancestral origins of the PhNPs used in this study, that is East Asia (haplotype D2), Southeast Asia (haplotype D7), and the Cordillera clade (Banayo et al., 2023). The initial ancestral polymorphisms between the D2 and D7 clades could explain the two initial clusters (*k* = 2) differentiating the NL and SLV regions, respectively (Figure 4). We previously hypothesized that the pigs of the D7 clade originated from the D2 clade via a genetic bottleneck event upon dispersal to Southeast Asia (Banayo, 2023). Thereafter, upon dispersal to various provinces in the Philippines, members of each clade formed further population structure via genetic drift (i.e., Q, M, and S of the D7 clade) due to the varied agroclimatic conditions and management practices of each province.

Contrary to local expectations, admixtures between native and transboundary breeds were generally low, confirming the historical disregard of native pigs by income‐driven production systems. Crossbreeding of native and transboundary breeds is widespread in Asia due to the pressures of higher productivity leading to loss of indigenous breeds (Charoensook et al., 2019; Tisdel, 2003; Zhang et al., 2016, 2018). Thus, the PhNP remains sufficiently differentiated both from transboundary breeds and from each other (Table 4, Figures 3 and 4). This finding implies the possibility of heterosis by crossbreeding with transboundary breeds and even with other PhNP populations. Supporting this view, an SL native pig was improved through crossbreeding with the Berkshire pig in the early 1900s, producing *Berkjala*, which is now extinct (Arboleda et al., 2003; Guiam, 2016).

Among the PhNP populations, the B population was the most differentiated according to the AMOVA results (Table 3). The high variance (*F*rt: 18%) between highland and lowland populations supports a long period of divergence, according to the assumptions of the SMM (Balloux & Goudet, 2002; Hardy et al., 2003; Li et al., 2017). However, their current low *Ne* elevates the risk of genetic erosion, similar to the related Lanyu pig of Taiwan (Chang et al., 2009; Wu et al., 2007). The Lanyu pig was previously reported to experience an increased rate of inbreeding and loss of heterozygosity (Chang et al., 2009), despite Lanyu islets being a potential domestication center (Li et al., 2017). Ethnic communities in the Cordillera region have long used the native pig in rituals and festivities, documented as early as 1902 in a folk epic called Hudhud (Lewis, 1992; Manuel, 1963). Genomic studies have shown that the Cordillerans themselves arrived in the Philippines much earlier than the arrival of agriculture (Larena et al., 2021). Prioritizing the conservation of the B population is thus a national concern not only to maintain ecological balance but also to protect indigenous and local knowledge, a progressive approach known as biocultural conservation (Llamazares et al., 2021). Understanding indigenous ways of life can inform modern society regarding the sustainable utilization of animal genetic resources (Llamazares et al., 2021).

Despite K being in the same administrative region as B (Cordillera) and having indigenous communities with their own rituals and traditions, the pig population in K had a high effective population size (*Ne*: 125.9), presumably due to introgression of wild pig alleles via interspecific hybridization. Having a high *Ne* is beneficial in that it increases the fitness of organisms (Chen et al., 2018). We previously identified an interspecific hybrid in the K population, but its ancestry could not be resolved with mtDNA D‐loop analysis (Banayo et al., 2023). New methods have been proposed that might achieve this aim (Lorenzini et al., 2020). In addition to the benefits of having high *Ne*, hybridization is known to introduce novel adaptive variations and further increase fitness (Adavoudi & Pilot, 2022). In fact, hybridization in French wild boar revealed adaptive introgression from domestic pigs (Mary et al., 2022). Our study suggests that a high *Ne* in native pigs can be achieved by hybridization with wild pigs, thus necessitating the comanagement of PhNPs and Philippine wild pigs.

Finally, this study showed that the B, I, Q, M, and S populations (*Ne* values <50) are immediate conservation priorities (Table 2). A minimum effective population size of 50–500 is recommended to maintain short‐term population viability, with the ultimate goal of achieving an effective population size >5000 to ensure long‐term viability (Franklin, 1980; Franklin & Frankham, 1998; Lynch & Lande, 1998). Therefore, all PhNP populations (*Ne* values <500) must be conserved to facilitate long‐term viability. Additionally, PhNP is an important repository of genetic variation, as shown by its high allele count (*Na*: 7.771) and heterozygosity (*Ho*: 0.737), thereby further warranting investments in their conservation. This study emphasized the importance of PhNP as a repository of genetic variation, primarily for adaptation traits. Further studies on the genetic basis of adaptation of the PhNP may be explored. Last, due to the economic advantage of using transboundary breeds, we recommend incentivizing native pig farming by funding agrobiodiversity conservation services to improve the sustainability of in situ or on‐farm conservation of the PhNP (Marsoner et al., 2018; Narloch et al., 2011; Wainwright et al., 2019).

## 5 CONCLUSION

This study shows the high genetic diversity of the PhNP and identifies six genetically distinct PhNP populations, of which the B, I, Q, M, and S populations are conservation priorities. Furthermore, this study provides a basis for both future hybridization studies between PhNP and wild pigs, and the assessment of the impact of ASF on PhNP genetic diversity.

## 6 RECOMMENDATIONS

We propose harnessing the diversity of the PhNP through crossbreeding (among PhNP populations or between the PhNP and transboundary breeds) to address the needs of modern farmers for a faster‐growing native pig while systematically avoiding population admixture. On the other hand, indigenous communities that raise PhNPs for cultural traditions, rather than income, must be incentivized to increase private benefits for utilizing PhNPs and subsequently sustaining on‐farm conservation. Since the majority are smallholder swine farms in the Philippines (PSA, 2022), a large community to perform agroecological services by utilizing PhNP remains to be tapped. Finally, we recommend continuous monitoring of PhNP diversity across different time points to assess the impact of interventions.

## AUTHOR CONTRIBUTIONS

**Joy B.Banayo:** Conceptualization (lead); formal analysis (lead); funding acquisition (equal); investigation (lead); methodology (lead); project administration (lead); validation (lead); visualization (lead); writing – original draft (lead); writing – review and editing (lead). **Kathlyn Louise V. Manese:** Formal analysis (supporting); investigation (supporting); project administration (supporting); writing – review and editing (supporting). **Kaito O. Furusho:** Formal analysis (supporting); visualization (supporting); writing – original draft (supporting). **Agapita J. Salces:** Conceptualization (supporting); funding acquisition (supporting); project administration (supporting); supervision (supporting); writing – review and editing (supporting). **Takahiro Yamagata:** Conceptualization (supporting); funding acquisition (equal); project administration (supporting); supervision (lead); writing – review and editing (supporting).

## FUNDING INFORMATION

This work was supported by Nagoya University‐Asian Satellite Campus Institute (NU‐ASCI), the University of the Philippines (UP), and the Department of Science and Technology ‐ Philippine Council for Agriculture, Aquatic and Natural Resources Research and Development (DOST‐PCAARRD).

## CONFLICT OF INTEREST STATEMENT

The authors have no relevant financial or nonfinancial interests to disclose.

### OPEN RESEARCH BADGES

This article has earned an Open Data badge for making publicly available the digitally‐shareable data necessary to reproduce the reported results. The data is available at [https://doi.org/10.5061/dryad.b8gtht7gg].

## Supporting information


**Tables S1.** **–S3.**

## Data Availability

The data that support the findings of this study are openly available in Dryad at https://doi.org/10.5061/dryad.b8gtht7gg. https://datadryad.org/stash/share/2lNW3Q0lSGDPud7_fSvTTzvT0FMik_Go_hjlY6q0RWU.

## APPENDIX A

**TABLE A1:** Sampling sites of Philippine native pigs represent 34 municipalities, 14 provinces, and 5 administrative regions.

Municipality	Province	Administrative region	Island	Number of samples
Bokod	Benguet	CAR^a^	NL^b^	2
Itogon	Benguet	CAR	NL	2
La Trinidad	Benguet	CAR	NL	8
Sablan	Benguet	CAR	NL	2
Tublay	Benguet	CAR	NL	1
Bauko	Mt. Province	CAR	NL	5
not specified	Mt. Province	CAR	NL	2
Lubuagan	Kalinga	CAR	NL	3
Pasil	Kalinga	CAR	NL	2
Tabuk City	Kalinga	CAR	NL	11
Tanudan	Kalinga	CAR	NL	6
Tinglayan	Kalinga	CAR	NL	5
Cordon	Isabela	II	NL	4
Echague	Isabela	II	NL	3
Gamu	Isabela	II	NL	7
Mallig	Isabela	II	NL	8
Maddela	Quirino	II	NL	4
Nagtipunan	Quirino	II	NL	1
Bagabag	Nueva Vizcaya	II	NL	4
Bayombong	Nueva Vizcaya	II	NL	6
Diadi	Nueva Vizcaya	II	NL	2
Dupax Del Norte	Nueva Vizcaya	II	NL	2
Santa Fe	Nueva Vizcaya	II	NL	1
Tiaong	Quezon	IVA	SL^c^	25
Sta. Cruz	Marinduque	IVB	SL	3
Torrijos	Marinduque	IVB	SL	18
Balangkayan	Eastern Samar	VIII	V^d^	1
Borongan City	Eastern Samar	VIII	V	6
Can‐avid	Eastern Samar	VIII	V	1
Dolores	Eastern Samar	VIII	V	1
Llorente	Eastern Samar	VIII	V	1
San Julian	Eastern Samar	VIII	V	4
Lope de Vega	Northern Samar	VIII	V	1
Gamay	Northern Samar	VIII	V	1
Paranas	Samar (formerly Western Samar)	VIII	V	2
Alang‐alang	Leyte	VIII	V	1
Ormoc City	Leyte	VIII	V	1
TOTAL				157

^a^ Cordillera Administrative Region.

^b^ North Luzon.

^c^ South Luzon.

^d^ Visayas.

**TABLE A2:** Informativeness of each of the 20 microsatellite loci used in the analysis.

Marker	*n*	Allele size (bp)	*Na*	*Ea*	*I*	*Ho*	*He*	*F*	*PIC*	*F*is	*F*it	*F*st	*Nm*	Adj. *P*_HWE_
IGF1	17.727	184‐208	6.182	3.351	1.351	0.713	0.649	−0.130	0.803	−0.099	0.144	0.221	0.883	0.386
S0005	17.818	204‐272	8.909	4.445	1.701	0.773	0.746	−0.040	0.824	−0.037	0.098	0.130	1.673	0.232
S0026	17.818		4.545	2.266	0.999	0.525	0.521	−0.031	0.605	−0.009	0.228	0.235	0.814	0.435
S0090	17.727	230‐254	4.909	3.106	1.256	0.654	0.638	−0.041	0.746	−0.025	0.157	0.178	1.151	0.144
S0097	17.727	204‐242	6.727	4.258	1.536	0.710	0.707	−0.027	0.816	−0.005	0.152	0.156	1.350	0.150
S0155	17.818	140‐168	5.818	3.701	1.383	0.638	0.667	0.037	0.819	0.044	0.238	0.203	0.982	0.210
S0226	17.727	180‐214	7.636	4.249	1.616	0.727	0.738	0.014	0.831	0.015	0.136	0.122	1.798	0.063
S0228	16.727	218‐248	7.364	4.541	1.586	0.767	0.719	−0.088	0.809	−0.067	0.046	0.106	2.118	0.669
S0355	17.727	244‐270	6.455	3.637	1.327	0.627	0.611	−0.052	0.748	−0.026	0.122	0.144	1.488	0.166
Sw122	17.727	102‐142	7.182	4.660	1.623	0.754	0.741	−0.034	0.852	−0.016	0.129	0.143	1.500	0.161
Sw24	17.273	94‐138	7.545	4.421	1.581	0.723	0.715	−0.011	0.853	−0.011	0.166	0.175	1.181	0.002^a^
Sw240	17.727	90‐124	6.909	3.359	1.445	0.682	0.680	−0.008	0.786	−0.003	0.158	0.161	1.305	0.303
Sw2406	17.727	224‐260	6.273	3.556	1.333	0.644	0.622	−0.048	0.801	−0.036	0.198	0.226	0.858	0.517
Sw2410	17.727	92‐136	5.364	2.995	1.165	0.580	0.573	−0.040	0.701	−0.012	0.136	0.146	1.464	0.118
Sw632	17.455	146‐176	5.818	3.339	1.302	0.668	0.633	−0.080	0.788	−0.055	0.175	0.218	0.895	0.487
Sw72	17.727	94‐126	5.636	3.107	1.296	0.680	0.643	−0.070	0.715	−0.056	0.096	0.144	1.480	0.644
Sw830	17.455	168‐234	5.545	3.330	1.295	0.621	0.635	0.011	0.737	0.022	0.166	0.147	1.455	0.215
Sw857	17.727	138‐170	6.636	4.183	1.561	0.767	0.732	−0.052	0.795	−0.047	0.067	0.109	2.044	0.536
Sw936	17.636	86‐116	5.909	3.821	1.429	0.697	0.693	−0.029	0.810	−0.005	0.160	0.164	1.275	0.167
Swr1941	17.364	204‐224	5.182	3.126	1.248	0.667	0.639	−0.040	0.744	−0.045	0.152	0.188	1.077	0.739
Mean	17.618		6.327	3.673	1.402	0.681	0.665	−0.038	0.779	−0.024	0.146	0.166	1.339	

Abbreviations: Adj. *P*_HWE_, *p*‐value HWE by heterozygote deficiency method and adjusted using FDR; bp, base pairs; *Ea*, number of effective alleles; *F*, fixation index; *He*, expected heterozygosity; *Ho*, observed heterozygosity; *I*, Shannon's information index; *n*, sample size; *Na*, number of different alleles; *Nm*, effective number of migrants.

^a^ Significant at alpha = .05.

**TABLE A3:** Matrix of Nei's *D*_*A*_ genetic distance and the effective number of migrants (*Nm*) between populations.

Population	Benguet	Kalinga	Nueva Vizcaya	Isabela	Quezon	Marinduque	Samar	Berkshire	Large White	Landrace	Duroc
Benguet	–	5.202	8.666	5.661	3.273	3.834	3.728	0.837	1.582	1.338	0.942
Kalinga	0.156	–	4.877	6.085	2.269	2.536	2.687	0.733	1.088	1.114	0.806
Nueva Vizcaya	0.156	0.164	–	9.290	8.042	10.138	8.460	1.120	2.312	2.500	1.524
Isabela	0.179	0.152	0.146	–	3.557	4.324	3.824	0.872	1.524	1.562	1.202
Quezon	0.208	0.241	0.153	0.205	–	9.869	6.625	1.068	2.124	3.152	1.285
Marinduque	0.212	0.226	0.146	0.214	0.136	–	8.174	1.154	2.606	2.788	1.554
Samar	0.221	0.225	0.167	0.225	0.184	0.158	–	1.085	2.288	2.177	1.278
Berkshire	0.495	0.507	0.445	0.505	0.391	0.377	0.442	–	0.777	0.743	0.491
Large White	0.396	0.431	0.338	0.376	0.305	0.281	0.325	0.447	–	2.047	0.933
Landrace	0.452	0.448	0.353	0.413	0.280	0.299	0.371	0.443	0.278	–	0.832
Duroc	0.469	0.483	0.346	0.410	0.352	0.322	0.393	0.530	0.413	0.408	–

*Note*: Below diagonal: Nei's *D*_*A*_ genetic distance; above diagonal: effective number of migrants (*Nm*).
