# Supplementary material for: Genetic diversity and population structure analysis of Philippine native pigs highlight five priority populations for conservation
Source: Ecol Evol. 2023 Oct 31;13(11):e10618. doi: 10.1002/ece3.10618 (PMC10618572; doi:10.1002/ece3.10618)
Supplement: Supplementary file 1 — Tables S1. –S3. [file ECE3-13-e10618-s001.docx]

**Appendix Table 1.** Sampling sites of Philippine native pigs represent 34 municipalities, 14 provinces, and 5 administrative regions.

| **Municipality** | **Province** | **Administrative region** | **Island** | **Number of samples** |
| --- | --- | --- | --- | --- |
| Bokod | Benguet | CAR^1^ | NL^2^ | 2 |
| Itogon | Benguet | CAR | NL | 2 |
| La Trinidad | Benguet | CAR | NL | 8 |
| Sablan | Benguet | CAR | NL | 2 |
| Tublay | Benguet | CAR | NL | 1 |
| Bauko | Mt. Province | CAR | NL | 5 |
| not specified | Mt. Province | CAR | NL | 2 |
| Lubuagan | Kalinga | CAR | NL | 3 |
| Pasil | Kalinga | CAR | NL | 2 |
| Tabuk City | Kalinga | CAR | NL | 11 |
| Tanudan | Kalinga | CAR | NL | 6 |
| Tinglayan | Kalinga | CAR | NL | 5 |
| Cordon | Isabela | II | NL | 4 |
| Echague | Isabela | II | NL | 3 |
| Gamu | Isabela | II | NL | 7 |
| Mallig | Isabela | II | NL | 8 |
| Maddela | Quirino | II | NL | 4 |
| Nagtipunan | Quirino | II | NL | 1 |
| Bagabag | Nueva Vizcaya | II | NL | 4 |
| Bayombong | Nueva Vizcaya | II | NL | 6 |
| Diadi | Nueva Vizcaya | II | NL | 2 |
| Dupax Del Norte | Nueva Vizcaya | II | NL | 2 |
| Santa Fe | Nueva Vizcaya | II | NL | 1 |
| Tiaong | Quezon | IVA | SL^3^ | 25 |
| Sta. Cruz | Marinduque | IVB | SL | 3 |
| Torrijos | Marinduque | IVB | SL | 18 |
| Balangkayan | Eastern Samar | VIII | V^4^ | 1 |
| Borongan City | Eastern Samar | VIII | V | 6 |
| Can-avid | Eastern Samar | VIII | V | 1 |
| Dolores | Eastern Samar | VIII | V | 1 |
| Llorente | Eastern Samar | VIII | V | 1 |
| San Julian | Eastern Samar | VIII | V | 4 |
| Lope de Vega | Northern Samar | VIII | V | 1 |
| Gamay | Northern Samar | VIII | V | 1 |
| Paranas | Samar (formerly Western Samar) | VIII | V | 2 |
| Alang-alang | Leyte | VIII | V | 1 |
| Ormoc City | Leyte | VIII | V | 1 |
| **TOTAL** |  |  |  | **157** |
| ^1^Cordillera Administrative Region, ^2^North Luzon, ^3^South Luzon, ^4^Visayas | | |  |  |

**Appendix Table 2.** Informativeness of each of the 20 microsatellite loci used in the analysis.

| **Marker** | ***n*** | **Allele size (bp)** | ***Na*** | ***Ea*** | ***I*** | ***Ho*** | ***He*** | ***F*** | ***PIC*** | ***Fis*** | ***Fit*** | ***Fst*** | ***Nm*** | **Adj. *P_HWE_*** |
| --- | --- | --- | --- | --- | --- | --- | --- | --- | --- | --- | --- | --- | --- | --- |
| IGF1 | 17.727 | 204-272 | 6.182 | 3.351 | 1.351 | 0.713 | 0.649 | -0.130 | 0.803 | -0.099 | 0.144 | 0.221 | 0.883 | 0.386 |
| S0005 | 17.818 | 140-168 | 8.909 | 4.445 | 1.701 | 0.773 | 0.746 | -0.040 | 0.824 | -0.037 | 0.098 | 0.130 | 1.673 | 0.210 |
| S0026 | 17.818 | 88-106 | 4.545 | 2.266 | 0.999 | 0.525 | 0.521 | -0.031 | 0.605 | -0.009 | 0.228 | 0.235 | 0.814 | 0.435 |
| S0090 | 17.727 | 244-270 | 4.909 | 3.106 | 1.256 | 0.654 | 0.638 | -0.041 | 0.746 | -0.025 | 0.157 | 0.178 | 1.151 | 0.118 |
| S0097 | 17.727 | 168-234 | 6.727 | 4.258 | 1.536 | 0.710 | 0.707 | -0.027 | 0.816 | -0.005 | 0.152 | 0.156 | 1.350 | 0.150 |
| S0155 | 17.818 | 92-136 | 5.818 | 3.701 | 1.383 | 0.638 | 0.667 | 0.037 | 0.819 | 0.044 | 0.238 | 0.203 | 0.982 | 0.210 |
| S0226 | 17.727 | 204-224 | 7.636 | 4.249 | 1.616 | 0.727 | 0.738 | 0.014 | 0.831 | 0.015 | 0.136 | 0.122 | 1.798 | 0.063 |
| S0228 | 16.727 | 146-176 | 7.364 | 4.541 | 1.586 | 0.767 | 0.719 | -0.088 | 0.809 | -0.067 | 0.046 | 0.106 | 2.118 | 0.669 |
| S0355 | 17.727 | 94-138 | 6.455 | 3.637 | 1.327 | 0.627 | 0.611 | -0.052 | 0.748 | -0.026 | 0.122 | 0.144 | 1.488 | 0.118 |
| Sw122 | 17.727 | 218-248 | 7.182 | 4.660 | 1.623 | 0.754 | 0.741 | -0.034 | 0.852 | -0.016 | 0.129 | 0.143 | 1.500 | 0.150 |
| Sw24 | 17.273 | 156-190 | 7.545 | 4.421 | 1.581 | 0.723 | 0.715 | -0.011 | 0.853 | -0.011 | 0.166 | 0.175 | 1.181 | 0.002* |
| Sw240 | 17.727 | 86-116 | 6.909 | 3.359 | 1.445 | 0.682 | 0.680 | -0.008 | 0.786 | -0.003 | 0.158 | 0.161 | 1.305 | 0.303 |
| Sw2406 | 17.727 | 204-242 | 6.273 | 3.556 | 1.333 | 0.644 | 0.622 | -0.048 | 0.801 | -0.036 | 0.198 | 0.226 | 0.858 | 0.487 |
| Sw2410 | 17.727 | 138-170 | 5.364 | 2.995 | 1.165 | 0.580 | 0.573 | -0.040 | 0.701 | -0.012 | 0.136 | 0.146 | 1.464 | 0.118 |
| Sw632 | 17.455 | 102-142 | 5.818 | 3.339 | 1.302 | 0.668 | 0.633 | -0.080 | 0.788 | -0.055 | 0.175 | 0.218 | 0.895 | 0.487 |
| Sw72 | 17.727 | 224-260 | 5.636 | 3.107 | 1.296 | 0.680 | 0.643 | -0.070 | 0.715 | -0.056 | 0.096 | 0.144 | 1.480 | 0.644 |
| Sw830 | 17.455 | 184-208 | 5.545 | 3.330 | 1.295 | 0.621 | 0.635 | 0.011 | 0.737 | 0.022 | 0.166 | 0.147 | 1.455 | 0.210 |
| Sw857 | 17.727 | 90-124 | 6.636 | 4.183 | 1.561 | 0.767 | 0.732 | -0.052 | 0.795 | -0.047 | 0.067 | 0.109 | 2.044 | 0.536 |
| Sw936 | 17.636 | 230-254 | 5.909 | 3.821 | 1.429 | 0.697 | 0.693 | -0.029 | 0.810 | -0.005 | 0.160 | 0.164 | 1.275 | 0.150 |
| Swr1941 | 17.364 | 180-214 | 5.182 | 3.126 | 1.248 | 0.667 | 0.639 | -0.040 | 0.744 | -0.045 | 0.152 | 0.188 | 1.077 | 0.739 |
| **Mean** | **17.618** | 94-126 | **6.327** | **3.673** | **1.402** | **0.681** | **0.665** | **-0.038** | **0.779** | **-0.024** | **0.146** | **0.166** | **1.339** |  |

*n* – sample size, bp- base pairs, *Na* – number of different alleles, *Ea* – number of effective alleles, *I* – Shannon’s information index, *Ho* – observed heterozygosity, *He* – expected heterozygosity, *F* – fixation index, *Nm* – effective number of migrants, Adj. *P_HWE_* – *p*-value HWE H1 = Heterozygote deficiency, adjusted using FDR, *significant at alpha 0.05

**Appendix Table 3.** Matrix of Nei’s *D_A_* genetic distance and the effective number of migrants (*Nm*) between populations.

| **Population** | **Benguet** | **Kalinga** | **Nueva Vizcaya** | **Isabela** | **Quezon** | **Marinduque** | **Samar** | **Berkshire** | **Large White** | **Landrace** | **Duroc** |
| --- | --- | --- | --- | --- | --- | --- | --- | --- | --- | --- | --- |
| Benguet | -- | 5.202 | 8.666 | 5.661 | 3.273 | 3.834 | 3.728 | 0.837 | 1.582 | 1.338 | 0.942 |
| Kalinga | 0.156 | -- | 4.877 | 6.085 | 2.269 | 2.536 | 2.687 | 0.733 | 1.088 | 1.114 | 0.806 |
| Nueva Vizcaya | 0.156 | 0.164 | -- | 9.290 | 8.042 | 10.138 | 8.460 | 1.120 | 2.312 | 2.500 | 1.524 |
| Isabela | 0.179 | 0.152 | 0.146 | -- | 3.557 | 4.324 | 3.824 | 0.872 | 1.524 | 1.562 | 1.202 |
| Quezon | 0.208 | 0.241 | 0.153 | 0.205 | -- | 9.869 | 6.625 | 1.068 | 2.124 | 3.152 | 1.285 |
| Marinduque | 0.212 | 0.226 | 0.146 | 0.214 | 0.136 | -- | 8.174 | 1.154 | 2.606 | 2.788 | 1.554 |
| Samar | 0.221 | 0.225 | 0.167 | 0.225 | 0.184 | 0.158 | -- | 1.085 | 2.288 | 2.177 | 1.278 |
| Berkshire | 0.495 | 0.507 | 0.445 | 0.505 | 0.391 | 0.377 | 0.442 | -- | 0.777 | 0.743 | 0.491 |
| Large White | 0.396 | 0.431 | 0.338 | 0.376 | 0.305 | 0.281 | 0.325 | 0.447 | -- | 2.047 | 0.933 |
| Landrace | 0.452 | 0.448 | 0.353 | 0.413 | 0.280 | 0.299 | 0.371 | 0.443 | 0.278 | -- | 0.832 |
| Duroc | 0.469 | 0.483 | 0.346 | 0.410 | 0.352 | 0.322 | 0.393 | 0.530 | 0.413 | 0.408 | -- |

Below diagonal: Nei’s *D_A_* genetic distance; above diagonal: effective number of migrants (*Nm*)

**Supplementary Table 1.** Fisher’s genotypic linkage disequilibrium (LD) test across 7 native pig populations revealed 131 locus pairs in LD.

| **Adjusted *p-*value** | **IGF1** | **S0005** | **S0026** | **S0090** | **S0097** | **S0155** | **S0226** | **S0228** | **S0355** | **Sw122** | **Sw24** | **Sw240** | **Sw2406** | **Sw2410** | **Sw632** | **Sw72** | **Sw830** | **Sw857** | **Sw936** | **Swr1941** |
| --- | --- | --- | --- | --- | --- | --- | --- | --- | --- | --- | --- | --- | --- | --- | --- | --- | --- | --- | --- | --- |
| IGF1 | - |  |  |  |  |  |  |  |  |  |  |  |  |  |  |  |  |  |  |  |
| S0005 | **0.000** | - |  |  |  |  |  |  |  |  |  |  |  |  |  |  |  |  |  |  |
| S0026 | 0.713 | 0.063 | - |  |  |  |  |  |  |  |  |  |  |  |  |  |  |  |  |  |
| S0090 | 0.197 | 0.172 | **0.020** | - |  |  |  |  |  |  |  |  |  |  |  |  |  |  |  |  |
| S0097 | **0.002** | 0.055 | 0.060 | **0.000** | - |  |  |  |  |  |  |  |  |  |  |  |  |  |  |  |
| S0155 | 0.714 | **0.000** | **0.003** | **0.005** | **0.037** | - |  |  |  |  |  |  |  |  |  |  |  |  |  |  |
| S0226 | 0.080 | **0.000** | **0.000** | **0.010** | **0.000** | 0.097 | - |  |  |  |  |  |  |  |  |  |  |  |  |  |
| S0228 | 0.342 | 0.071 | **0.024** | 0.794 | **0.007** | 0.052 | 0.097 | - |  |  |  |  |  |  |  |  |  |  |  |  |
| S0355 | 0.140 | **0.000** | **0.028** | 0.057 | **0.000** | **0.000** | **0.000** | **0.027** | **-** |  |  |  |  |  |  |  |  |  |  |  |
| Sw122 | **0.000** | **0.000** | 0.158 | 0.355 | **0.017** | **0.000** | **0.000** | **0.011** | **0.000** | - |  |  |  |  |  |  |  |  |  |  |
| Sw24 | 0.158 | **0.014** | **0.007** | **0.018** | 0.102 | **0.023** | 0.134 | **0.037** | **0.036** | **0.000** | - |  |  |  |  |  |  |  |  |  |
| Sw240 | **0.022** | **0.004** | 0.143 | 0.073 | **0.001** | **0.044** | **0.027** | **0.002** | **0.010** | 0.121 | **0.007** | - |  |  |  |  |  |  |  |  |
| Sw2406 | **0.005** | **0.000** | **0.022** | 0.081 | **0.018** | **0.000** | **0.000** | 0.390 | 0.157 | **0.000** | **0.000** | **0.020** | - |  |  |  |  |  |  |  |
| Sw2410 | 0.212 | **0.000** | 0.671 | 0.108 | 0.080 | 0.071 | **0.000** | 0.072 | **0.014** | **0.033** | **0.000** | **0.037** | **0.000** | - |  |  |  |  |  |  |
| Sw632 | 0.183 | **0.000** | 0.097 | 0.205 | **0.000** | **0.022** | **0.000** | 0.069 | **0.000** | **0.000** | **0.000** | **0.010** | **0.000** | 0.050 | - |  |  |  |  |  |
| Sw72 | **0.000** | **0.000** | **0.007** | 0.051 | **0.004** | **0.017** | **0.000** | **0.007** | **0.000** | **0.000** | **0.000** | **0.006** | **0.005** | 0.102 | **0.000** | - |  |  |  |  |
| Sw830 | 0.227 | **0.000** | **0.010** | 0.391 | **0.030** | 0.163 | **0.007** | **0.009** | **0.000** | **0.000** | **0.010** | **0.000** | **0.000** | **0.001** | **0.005** | **0.015** | - |  |  |  |
| Sw857 | 0.079 | **0.000** | 0.017 | 0.438 | **0.003** | **0.001** | **0.000** | **0.000** | 0.069 | **0.000** | **0.000** | **0.018** | 0.088 | 0.247 | **0.000** | **0.000** | **0.000** | - |  |  |
| Sw936 | 0.102 | 0.177 | 0.245 | 0.574 | **0.010** | 0.055 | **0.001** | 0.140 | **0.010** | **0.000** | 0.073 | **0.007** | 0.109 | 0.123 | **0.015** | **0.063** | **0.001** | **0.007** | - |  |
| Swr1941 | **0.000** | **0.000** | **0.000** | 0.249 | 0.024 | **0.001** | **0.004** | 0.072 | **0.000** | **0.000** | **0.000** | **0.037** | **0.000** | **0.000** | **0.005** | **0.001** | **0.000** | **0.000** | **0.000** | - |

*Adjusted using FDR, this test was implemented in GENEPOP (Rousset, 2008), bold – significant at alpha = 0.05

**Supplementary Table 2.** Genotypic linkage disequilibrium test by subpopulation.

|  | **Adjusted *p*-value** | | | | | | | |
| --- | --- | --- | --- | --- | --- | --- | --- | --- |
| **Locus Pair** | **B** | **K** | **N** | **I** | **Q** | **M** | **S** | **ALL** |
| Sample size | 22 | 27 | 20 | 23 | 25 | 20 | 20 | 157 |
| IGF1 - S0005 | **0.00** | 1.12 | 0.71 | 1.02 | 0.36 | 0.49 | 0.51 | 0.00 |
| IGF1 - S0026 | 0.56 | 1.40 | 0.73 | 0.99 | 0.57 | 0.90 | 0.34 | 0.71 |
| IGF1 - S0090 | **0.05** | 1.32 | 1.45 | 1.01 | 0.45 | 0.91 | 0.09 | 0.20 |
| IGF1 - S0097 | **0.02** | 1.57 | 0.22 | 1.05 | **0.03** | 0.69 | 0.31 | 0.00 |
| IGF1 - S0155 | 0.39 | 1.14 | 1.07 | 1.05 | 0.28 | 0.50 | 0.55 | 0.71 |
| IGF1 - S0226 | 0.09 | 1.13 | 1.45 | 0.98 | 0.14 | 0.88 | **0.04** | 0.08 |
| IGF1 - S0228 | 0.59 | 1.16 | 0.49 | 1.00 | 0.28 | 0.54 | 0.33 | 0.34 |
| IGF1 - S0355 | 0.32 | 1.35 | 1.45 | 0.48 | 0.74 | 0.36 | 0.27 | 0.14 |
| IGF1 - Sw122 | **0.00** | 1.43 | 0.86 | 1.05 | 0.26 | 0.79 | 0.32 | 0.00 |
| IGF1 - Sw24 | **0.02** | 1.13 | 0.78 | 0.91 | 0.29 | 0.77 | 0.47 | 0.16 |
| IGF1 - Sw240 | 0.12 | 1.15 | 0.25 | 0.82 | 0.26 | 0.60 | 0.07 | 0.02 |
| IGF1 - Sw2406 | **0.01** | 1.17 | 0.89 | 0.73 | 0.34 | 0.60 | **0.03** | 0.01 |
| IGF1 - Sw2410 | 0.09 | 1.11 | 0.89 | 0.92 | 0.40 | 0.55 | 0.21 | 0.21 |
| IGF1 - Sw632 | **0.01** | 1.30 | 1.10 | 1.05 | 0.21 | 1.06 | 0.42 | 0.18 |
| IGF1 - Sw72 | **0.00** | 1.16 | 1.10 | 0.95 | 0.32 | 0.37 | 0.08 | 0.00 |
| IGF1 - Sw830 | **0.03** | 1.16 | 1.45 | 0.73 | 0.45 | 0.65 | 0.26 | 0.23 |
| IGF1 - Sw857 | **0.02** | 1.11 | 0.89 | 1.06 | 0.28 | 0.46 | 0.73 | 0.08 |
| IGF1 - Sw936 | **0.01** | 1.24 | 1.45 | 0.92 | 0.21 | 1.13 | 0.26 | 0.10 |
| IGF1 - Swr1941 | **0.00** | 1.15 | 0.70 | 0.47 | **0.04** | 0.41 | 0.91 | 0.00 |
| S0005 - S0026 | 0.18 | 1.28 | 1.06 | 0.60 | 0.11 | 0.69 | 0.10 | 0.06 |
| S0005 - S0090 | **0.03** | 1.16 | 1.45 | 0.68 | 0.20 | 1.13 | 0.35 | 0.17 |
| S0005 - S0097 | 0.17 | 1.14 | 1.45 | 0.54 | **0.03** | 0.54 | 0.26 | 0.05 |
| S0005 - S0155 | **0.02** | 1.57 | 0.52 | 0.78 | **0.04** | 0.43 | **0.02** | 0.00 |
| S0005 - S0226 | **0.03** | 1.22 | 1.45 | 0.34 | **0.00** | 1.13 | 0.33 | 0.00 |
| S0005 - S0228 | 0.57 | 1.44 | 0.74 | 1.05 | 0.08 | 0.37 | 0.18 | 0.07 |
| S0005 - S0355 | 0.25 | 1.16 | 1.45 | 1.00 | 0.13 | 0.52 | **0.00** | 0.00 |
| S0005 - Sw122 | **0.00** | 1.14 | 0.73 | 0.38 | **0.00** | 0.41 | 0.31 | 0.00 |
| S0005 - Sw24 | **0.01** | 1.22 | 0.67 | 0.92 | 0.05 | 1.13 | 0.21 | 0.01 |
| S0005 - Sw240 | 0.18 | 1.18 | 0.66 | 1.01 | **0.00** | 0.65 | 0.39 | 0.00 |
| S0005 - Sw2406 | **0.00** | 1.21 | 1.45 | 0.95 | 0.13 | 0.49 | 0.31 | 0.00 |
| S0005 - Sw2410 | **0.00** | 1.17 | 0.63 | 1.05 | 0.26 | 0.64 | 0.48 | 0.00 |
| S0005 - Sw632 | **0.01** | 0.23 | 0.77 | 0.91 | **0.00** | 1.13 | 0.32 | 0.00 |
| S0005 - Sw72 | **0.00** | 1.25 | 1.45 | 1.05 | **0.04** | 0.42 | 0.40 | 0.00 |
| S0005 - Sw830 | **0.00** | 1.21 | 1.45 | 0.91 | 0.23 | **0.00** | **0.04** | 0.00 |
| S0005 - Sw857 | **0.01** | 1.16 | 0.90 | 0.92 | **0.00** | 0.42 | 0.32 | 0.00 |
| S0005 - Sw936 | **0.01** | 1.15 | 1.45 | 0.54 | 0.26 | 1.13 | 1.00 | 0.18 |
| S0005 - Swr1941 | **0.00** | 1.01 | 1.45 | 0.47 | 0.14 | 0.49 | 0.37 | 0.00 |
| S0026 - S0090 | **0.03** | 1.23 | 0.66 | 0.36 | 0.27 | 0.73 | 0.27 | 0.02 |
| S0026 - S0097 | 0.08 | 1.17 | 0.90 | 0.60 | 0.08 | 0.79 | 0.22 | 0.06 |
| S0026 - S0155 | **0.01** | 1.16 | 0.42 | 0.49 | 0.13 | 0.61 | 0.31 | 0.00 |
| S0026 - S0226 | 0.17 | 1.15 | 0.75 | 0.91 | **0.00** | 0.43 | 0.25 | 0.00 |
| S0026 - S0228 | 0.69 | 1.60 | 0.52 | 0.69 | 0.17 | 0.44 | 0.18 | 0.02 |
| S0026 - S0355 | 0.29 | 1.41 | 0.88 | 1.06 | 0.11 | 0.43 | **0.04** | 0.03 |
| S0026 - Sw122 | 0.14 | 1.15 | 0.67 | 0.51 | 0.13 | 0.78 | 0.68 | 0.16 |
| S0026 - Sw24 | **0.00** | 1.15 | 0.88 | 0.52 | 0.28 | 0.89 | **0.04** | 0.01 |
| S0026 - Sw240 | 0.30 | 1.15 | 0.72 | 0.99 | 0.13 | 0.69 | 0.12 | 0.14 |
| S0026 - Sw2406 | **0.02** | 1.55 | 1.35 | 0.46 | 0.21 | 0.49 | 0.26 | 0.02 |
| S0026 - Sw2410 | 0.16 | 1.15 | 1.45 | 1.06 | 0.21 | 0.62 | 0.72 | 0.67 |
| S0026 - Sw632 | **0.04** | 1.13 | 1.45 | 0.74 | 0.26 | 0.98 | 0.07 | 0.10 |
| S0026 - Sw72 | **0.01** | 1.19 | 1.45 | 0.46 | 0.08 | 0.60 | 0.10 | 0.01 |
| S0026 - Sw830 | 0.28 | 1.36 | 0.15 | 0.79 | 0.21 | 0.42 | 0.21 | 0.01 |
| S0026 - Sw857 | **0.02** | 1.07 | 0.69 | 0.78 | 0.27 | 0.60 | 0.30 | 0.02 |
| S0026 - Sw936 | 0.50 | 1.38 | 0.41 | 0.68 | 0.26 | 0.91 | 0.55 | 0.25 |
| S0026 - Swr1941 | 0.06 | 0.45 | 0.52 | 0.91 | 0.07 | 0.41 | 0.27 | 0.00 |
| S0090 - S0097 | **0.00** | 1.29 | 1.45 | 0.67 | 0.13 | 1.13 | **0.03** | 0.00 |
| S0090 - S0155 | **0.01** | 1.16 | 1.08 | 0.95 | 0.07 | 0.73 | **0.02** | 0.00 |
| S0090 - S0226 | 0.10 | 1.17 | 0.79 | 0.92 | 0.45 | 0.06 | 0.23 | 0.01 |
| S0090 - S0228 | 1.00 | 1.16 | 1.02 | 1.05 | 0.11 | 1.13 | 0.38 | 0.79 |
| S0090 - S0355 | **0.03** | 1.49 | 1.45 | 0.69 | 0.31 | 0.40 | 0.24 | 0.06 |
| S0090 - Sw122 | 0.13 | 1.26 | 1.45 | 0.69 | 0.51 | 1.13 | 0.28 | 0.35 |
| S0090 - Sw24 | **0.01** | 1.42 | 1.45 | 0.34 | 0.29 | 0.73 | 0.35 | 0.02 |
| S0090 - Sw240 | 0.68 | 1.11 | 1.45 | 0.67 | 0.07 | 0.97 | **0.03** | 0.07 |
| S0090 - Sw2406 | 0.28 | 1.24 | 0.93 | 0.59 | 0.15 | 0.49 | 0.27 | 0.08 |
| S0090 - Sw2410 | **0.02** | 1.30 | 1.45 | 1.02 | 0.20 | 0.49 | 0.32 | 0.11 |
| S0090 - Sw632 | 0.13 | 1.07 | 1.45 | 0.58 | 0.53 | 0.72 | 0.40 | 0.21 |
| S0090 - Sw72 | 0.08 | 1.43 | 1.45 | 0.92 | 0.14 | 0.39 | 0.22 | 0.05 |
| S0090 - Sw830 | 0.20 | 1.46 | 0.91 | 0.78 | 0.43 | 1.13 | 0.23 | 0.39 |
| S0090 - Sw857 | 0.19 | 1.15 | 1.31 | 0.92 | 0.29 | 1.13 | 0.11 | 0.44 |
| S0090 - Sw936 | 0.14 | 1.14 | 1.45 | 0.87 | 0.81 | 0.75 | 0.18 | 0.57 |
| S0090 - Swr1941 | 0.09 | 1.20 | 1.45 | 0.93 | 0.47 | 0.49 | 0.22 | 0.25 |
| S0097 - S0155 | 0.09 | 1.16 | 0.88 | 0.22 | 0.38 | 0.47 | 0.40 | 0.04 |
| S0097 - S0226 | 0.09 | 1.17 | 0.75 | 0.79 | **0.00** | 1.13 | **0.00** | 0.00 |
| S0097 - S0228 | 0.12 | 1.16 | 0.21 | 0.69 | 0.09 | 0.50 | 0.11 | 0.01 |
| S0097 - S0355 | **0.00** | 1.54 | 1.45 | 0.78 | 0.08 | 0.69 | **0.00** | 0.00 |
| S0097 - Sw122 | 0.06 | 1.22 | 1.45 | 0.81 | 0.17 | 0.49 | 0.07 | 0.02 |
| S0097 - Sw24 | 0.08 | 1.16 | 1.45 | 0.41 | 0.09 | 1.13 | 0.33 | 0.10 |
| S0097 - Sw240 | 0.14 | 1.02 | 0.61 | 0.83 | **0.03** | 0.60 | **0.03** | 0.00 |
| S0097 - Sw2406 | **0.01** | 1.15 | 0.80 | 0.48 | 0.29 | 0.87 | 0.07 | 0.02 |
| S0097 - Sw2410 | 0.07 | 1.16 | 0.74 | 0.67 | 0.38 | 0.90 | 0.07 | 0.08 |
| S0097 - Sw632 | **0.00** | 1.28 | 0.92 | 0.35 | 0.14 | 1.06 | **0.04** | 0.00 |
| S0097 - Sw72 | **0.04** | 1.20 | 0.90 | 0.36 | 0.15 | 0.50 | 0.09 | 0.00 |
| S0097 - Sw830 | **0.03** | 1.16 | 0.89 | 0.47 | 0.64 | 0.91 | **0.03** | 0.03 |
| S0097 - Sw857 | 0.08 | 1.08 | 1.08 | 0.46 | 0.06 | 0.48 | 0.08 | 0.00 |
| S0097 - Sw936 | 0.20 | 1.49 | 0.31 | 0.92 | **0.04** | 1.13 | 0.07 | 0.01 |
| S0097 - Swr1941 | 0.07 | 1.28 | 0.53 | 1.05 | 0.35 | 0.49 | 0.22 | 0.02 |
| S0155 - S0226 | **0.03** | 1.15 | 0.91 | 0.92 | 0.07 | 0.64 | 0.52 | 0.10 |
| S0155 - S0228 | 1.00 | 1.16 | 0.17 | 0.68 | 0.14 | 0.41 | 0.48 | 0.05 |
| S0155 - S0355 | **0.00** | 1.51 | 0.80 | 0.47 | **0.01** | 0.46 | **0.04** | 0.00 |
| S0155 - Sw122 | 0.25 | 1.12 | 0.50 | 0.46 | **0.03** | 0.49 | **0.00** | 0.00 |
| S0155 - Sw24 | **0.03** | 1.12 | 0.51 | 0.48 | 0.24 | 1.13 | 0.21 | 0.02 |
| S0155 - Sw240 | 0.22 | 1.57 | 0.78 | 0.38 | 0.29 | 0.49 | 0.26 | 0.04 |
| S0155 - Sw2406 | **0.00** | 1.12 | 1.45 | 0.68 | 0.27 | 0.39 | 0.46 | 0.00 |
| S0155 - Sw2410 | **0.02** | 1.27 | 1.45 | 0.92 | 0.21 | 0.38 | 0.45 | 0.07 |
| S0155 - Sw632 | 0.13 | 1.60 | 1.09 | 0.48 | **0.04** | 0.89 | 0.18 | 0.02 |
| S0155 - Sw72 | 0.05 | 1.45 | 0.81 | 0.49 | 0.13 | 0.45 | 0.24 | 0.02 |
| S0155 - Sw830 | 0.10 | 1.34 | 0.99 | 0.79 | 0.64 | 0.45 | 0.18 | 0.16 |
| S0155 - Sw857 | **0.00** | 1.11 | 0.55 | 0.50 | 0.05 | 0.40 | 0.87 | 0.00 |
| S0155 - Sw936 | 0.18 | 1.36 | 0.22 | 0.48 | 0.23 | 1.13 | 0.37 | 0.05 |
| S0155 - Swr1941 | **0.02** | 0.82 | 0.18 | 0.92 | 0.26 | 0.43 | 0.26 | 0.00 |
| S0226 - S0228 | **0.03** | 1.16 | 0.72 | 0.92 | 0.23 | 1.13 | 0.12 | 0.10 |
| S0226 - S0355 | 0.15 | 1.16 | 1.45 | 0.92 | **0.01** | 0.38 | **0.00** | 0.00 |
| S0226 - Sw122 | **0.01** | 1.57 | 1.45 | 0.35 | **0.00** | 1.13 | 0.13 | 0.00 |
| S0226 - Sw24 | 0.06 | 1.19 | 1.45 | 0.67 | 0.17 | 0.72 | 0.26 | 0.13 |
| S0226 - Sw240 | 0.56 | 1.23 | 1.45 | 0.43 | **0.03** | 0.94 | 0.07 | 0.03 |
| S0226 - Sw2406 | **0.00** | 1.21 | 0.36 | 0.66 | 0.22 | 0.43 | **0.03** | 0.00 |
| S0226 - Sw2410 | **0.00** | 1.15 | 1.45 | 0.78 | 0.17 | 0.40 | **0.00** | 0.00 |
| S0226 - Sw632 | 0.12 | 1.19 | 0.84 | 0.91 | **0.03** | 0.40 | **0.02** | 0.00 |
| S0226 - Sw72 | **0.00** | 1.03 | 0.82 | 0.49 | **0.05** | 0.50 | 0.06 | 0.00 |
| S0226 - Sw830 | 0.06 | 1.49 | 1.45 | 0.78 | **0.01** | 1.13 | 0.10 | 0.01 |
| S0226 - Sw857 | **0.00** | 1.15 | 0.61 | 0.35 | **0.04** | 1.13 | 0.18 | 0.00 |
| S0226 - Sw936 | **0.01** | 1.20 | 0.66 | 0.79 | 0.06 | 0.40 | 0.11 | 0.00 |
| S0226 - Swr1941 | **0.02** | 1.14 | 0.75 | 0.41 | 0.06 | 0.45 | 0.26 | 0.00 |
| S0228 - S0355 | 0.08 | 1.16 | 0.74 | 1.07 | 0.65 | 0.51 | **0.01** | 0.03 |
| S0228 - Sw122 | **0.02** | 1.16 | 0.47 | 1.05 | 0.13 | 0.42 | 0.15 | 0.01 |
| S0228 - Sw24 | 0.07 | 1.16 | 0.31 | 0.78 | 0.10 | 0.51 | 0.61 | 0.04 |
| S0228 - Sw240 | 0.32 | 1.20 | 0.61 | 1.06 | 0.05 | 0.39 | **0.01** | 0.00 |
| S0228 - Sw2406 | 0.37 | 1.16 | 1.45 | 0.47 | 0.45 | 0.49 | 0.35 | 0.39 |
| S0228 - Sw2410 | 0.28 | 1.16 | 1.45 | 0.46 | **0.05** | 0.60 | 0.26 | 0.07 |
| S0228 - Sw632 | 0.32 | 1.16 | 0.96 | 0.81 | 0.07 | 0.60 | 0.07 | 0.07 |
| S0228 - Sw72 | **0.04** | 1.18 | 1.00 | 0.33 | 0.14 | 0.49 | 0.21 | 0.01 |
| S0228 - Sw830 | 0.06 | 1.45 | 0.91 | 0.47 | 0.25 | 0.39 | 0.11 | 0.01 |
| S0228 - Sw857 | 0.62 | 1.52 | 0.29 | 0.46 | **0.00** | 0.46 | 0.33 | 0.00 |
| S0228 - Sw936 | 0.42 | 1.16 | 0.26 | 0.79 | 0.16 | 1.13 | 0.31 | 0.14 |
| S0228 - Swr1941 | 0.72 | 1.16 | 0.16 | 0.95 | 0.09 | 0.49 | 0.73 | 0.07 |
| S0355 - Sw122 | 0.73 | 1.13 | 1.45 | 1.05 | **0.00** | 0.42 | 0.10 | 0.00 |
| S0355 - Sw24 | 0.20 | 1.16 | 0.64 | 0.47 | 0.07 | 0.44 | 0.31 | 0.04 |
| S0355 - Sw240 | 0.23 | 1.22 | 1.45 | 0.47 | 0.23 | 0.53 | **0.03** | 0.01 |
| S0355 - Sw2406 | 0.46 | 1.27 | 1.45 | 1.01 | 0.72 | 0.42 | 0.07 | 0.16 |
| S0355 - Sw2410 | **0.05** | 1.16 | 1.45 | 0.92 | 0.26 | 0.16 | 0.07 | 0.01 |
| S0355 - Sw632 | 0.18 | 1.14 | 1.45 | 0.94 | 0.05 | 0.38 | **0.00** | 0.00 |
| S0355 - Sw72 | 0.45 | 1.12 | 1.45 | 0.92 | **0.00** | 0.38 | **0.04** | 0.00 |
| S0355 - Sw830 | 0.06 | 1.17 | 0.87 | 0.36 | 0.08 | 0.25 | **0.00** | 0.00 |
| S0355 - Sw857 | 0.08 | 1.16 | 0.96 | 0.89 | 0.10 | 0.78 | 0.07 | 0.07 |
| S0355 - Sw936 | 0.23 | 1.41 | 0.71 | 1.02 | **0.01** | 0.82 | 0.05 | 0.01 |
| S0355 - Swr1941 | 0.25 | 1.14 | 0.77 | 0.92 | 0.26 | **0.00** | **0.03** | 0.00 |
| Sw122 - Sw24 | **0.00** | 0.41 | 0.72 | 0.48 | **0.05** | 0.60 | 0.33 | 0.00 |
| Sw122 - Sw240 | 0.25 | 1.24 | 0.55 | 0.79 | 0.34 | 0.78 | 0.26 | 0.12 |
| Sw122 - Sw2406 | **0.00** | 1.16 | 0.78 | 0.75 | 0.19 | 0.72 | 0.16 | 0.00 |
| Sw122 - Sw2410 | 0.12 | 1.16 | 1.45 | 0.67 | 0.06 | 0.55 | 0.07 | 0.03 |
| Sw122 - Sw632 | **0.00** | 1.18 | 0.88 | 0.77 | **0.00** | 0.83 | **0.04** | 0.00 |
| Sw122 - Sw72 | **0.00** | 1.12 | 1.45 | 1.05 | **0.04** | 0.72 | **0.00** | 0.00 |
| Sw122 - Sw830 | **0.01** | 1.16 | 0.06 | 0.81 | **0.05** | 0.75 | **0.00** | 0.00 |
| Sw122 - Sw857 | **0.00** | 1.17 | 0.50 | 0.91 | **0.00** | 0.60 | 0.34 | 0.00 |
| Sw122 - Sw936 | **0.00** | 1.33 | 0.88 | **0.00** | **0.00** | 1.13 | 0.39 | 0.00 |
| Sw122 - Swr1941 | **0.00** | 1.15 | 0.90 | 0.58 | **0.00** | 0.65 | 0.65 | 0.00 |
| Sw24 - Sw240 | **0.03** | 1.17 | 0.09 | 0.38 | 0.45 | 0.89 | 0.36 | 0.01 |
| Sw24 - Sw2406 | **0.00** | 1.16 | 1.45 | 0.35 | **0.03** | 1.13 | 0.26 | 0.00 |
| Sw24 - Sw2410 | **0.00** | 0.64 | 1.45 | 0.31 | **0.00** | 0.63 | 0.55 | 0.00 |
| Sw24 - Sw632 | **0.00** | 1.18 | 0.52 | 0.67 | **0.04** | 0.40 | 0.41 | 0.00 |
| Sw24 - Sw72 | **0.00** | 1.39 | 0.90 | 0.72 | 0.09 | 0.75 | 0.48 | 0.00 |
| Sw24 - Sw830 | 0.08 | 1.23 | 1.45 | 0.69 | **0.04** | 0.39 | 0.48 | 0.01 |
| Sw24 - Sw857 | **0.01** | 1.16 | 0.54 | 0.20 | 0.05 | 0.62 | 0.33 | 0.00 |
| Sw24 - Sw936 | **0.01** | 1.13 | 0.76 | 0.42 | 0.57 | 0.64 | 1.00 | 0.07 |
| Sw24 - Swr1941 | **0.00** | 1.18 | 0.80 | 0.57 | 0.18 | 0.73 | 0.48 | 0.00 |
| Sw240 - Sw2406 | **0.00** | 1.15 | 1.45 | 0.93 | 0.76 | 0.44 | 0.07 | 0.02 |
| Sw240 - Sw2410 | 0.22 | 1.15 | 1.45 | 0.53 | 0.06 | 0.78 | **0.04** | 0.04 |
| Sw240 - Sw632 | **0.00** | 1.15 | 1.03 | 0.93 | 0.33 | 0.39 | 0.11 | 0.01 |
| Sw240 - Sw72 | **0.01** | 1.24 | 1.09 | 0.73 | **0.04** | 0.79 | 0.07 | 0.01 |
| Sw240 - Sw830 | **0.00** | 1.39 | 1.03 | 0.91 | 0.07 | 0.45 | 0.18 | 0.00 |
| Sw240 - Sw857 | 0.07 | 1.21 | 0.67 | 0.92 | 0.11 | 0.40 | 0.21 | 0.02 |
| Sw240 - Sw936 | **0.03** | 1.13 | 0.90 | 0.46 | 0.80 | 0.41 | **0.03** | 0.01 |
| Sw240 - Swr1941 | 0.14 | 1.23 | 0.71 | 1.07 | **0.04** | 0.38 | 0.40 | 0.04 |
| Sw2406 - Sw2410 | **0.01** | 1.15 | 0.80 | 0.48 | **0.04** | 0.38 | **0.04** | 0.00 |
| Sw2406 - Sw632 | **0.00** | 1.17 | 0.94 | 0.10 | 0.26 | 0.74 | 0.21 | 0.00 |
| Sw2406 - Sw72 | **0.01** | 1.26 | 0.33 | 0.34 | 0.54 | 0.62 | 0.27 | 0.01 |
| Sw2406 - Sw830 | **0.00** | 1.16 | 1.45 | 0.33 | 0.19 | 0.92 | 0.23 | 0.00 |
| Sw2406 - Sw857 | **0.01** | 1.16 | 1.13 | 0.61 | 0.23 | 0.74 | 0.33 | 0.09 |
| Sw2406 - Sw936 | **0.04** | 1.14 | 1.45 | 0.57 | 0.26 | 0.79 | 0.16 | 0.11 |
| Sw2406 - Swr1941 | **0.00** | 1.42 | 1.45 | 0.92 | 0.20 | 0.38 | 0.61 | 0.00 |
| Sw2410 - Sw632 | 0.09 | 1.30 | 0.92 | 0.68 | 0.15 | 0.60 | 0.18 | 0.05 |
| Sw2410 - Sw72 | 0.18 | 1.44 | 0.52 | 1.05 | 0.57 | 0.49 | 0.12 | 0.10 |
| Sw2410 - Sw830 | **0.00** | 1.29 | 1.45 | 0.66 | 0.11 | 0.45 | 0.07 | 0.00 |
| Sw2410 - Sw857 | **0.04** | 1.16 | 1.06 | 0.68 | 0.28 | 0.90 | 0.31 | 0.25 |
| Sw2410 - Sw936 | **0.04** | 1.11 | 1.45 | 0.92 | 0.27 | 1.13 | 0.27 | 0.12 |
| Sw2410 - Swr1941 | **0.00** | 1.15 | 1.45 | 1.05 | **0.04** | **0.00** | 0.65 | 0.00 |
| Sw632 - Sw72 | **0.00** | 1.47 | 1.17 | 0.47 | 0.13 | 0.37 | **0.03** | 0.00 |
| Sw632 - Sw830 | **0.01** | 1.16 | 1.45 | 0.43 | 0.55 | 0.47 | 0.07 | 0.01 |
| Sw632 - Sw857 | **0.04** | 1.16 | 0.72 | 0.94 | **0.00** | 0.85 | **0.03** | 0.00 |
| Sw632 - Sw936 | **0.02** | 1.18 | 0.92 | 0.72 | 0.09 | 0.60 | 0.14 | 0.02 |
| Sw632 - Swr1941 | **0.01** | 1.15 | 1.08 | 0.47 | 0.07 | 0.45 | 0.33 | 0.00 |
| Sw72 - Sw830 | **0.01** | 1.16 | 1.45 | 0.68 | 0.71 | 0.65 | 0.07 | 0.02 |
| Sw72 - Sw857 | **0.01** | 1.33 | 1.10 | 0.09 | 0.36 | 0.48 | 0.07 | 0.00 |
| Sw72 - Sw936 | **0.00** | 1.16 | 1.45 | 1.01 | 0.26 | 0.78 | 0.25 | 0.06 |
| Sw72 - Swr1941 | **0.00** | 1.31 | 1.45 | 0.85 | 0.12 | 0.07 | 0.55 | 0.00 |
| Sw830 - Sw857 | **0.00** | 1.16 | 0.66 | 1.06 | 0.49 | 0.81 | **0.00** | 0.00 |
| Sw830 - Sw936 | **0.01** | 1.18 | 0.23 | 0.78 | 0.18 | 0.79 | **0.03** | 0.00 |
| Sw830 - Swr1941 | **0.00** | 1.27 | 0.94 | 1.06 | 0.06 | 0.42 | **0.00** | 0.00 |
| Sw857 - Sw936 | **0.00** | 1.12 | 0.62 | 0.80 | 0.07 | 0.69 | 0.33 | 0.01 |
| Sw857 - Swr1941 | **0.00** | 1.20 | 0.67 | 0.48 | 0.32 | 0.75 | **0.04** | 0.00 |
| Sw936 - Swr1941 | **0.02** | 1.12 | **0.00** | 1.05 | 0.23 | 0.41 | 0.52 | 0.00 |
| Number of locus pairs in LD | 107 | 0 | 1 | 1 | 45 | 3 | 40 | **131.00** |
| Percentage of total | 77% | 0% | 1% | 1% | 32% | 2% | 29% |  |

*Adjusted using FDR, this test was implemented in GENEPOP (Rousset, 2008)

**Supplementary Table 3.** Abbreviated table demonstrating results of linkage disequilibrium (LD) analysis on each pair of loci of Philippine native pig subpopulations.

| **Locus Pair** | **Number of Comparisons** | **Common Correlations** | **Chi-Square** | **Degrees of Freedom** | **Adjusted *p*-value** |
| --- | --- | --- | --- | --- | --- |
| IGF1 - S0005 | 157 | 0.06 | 343.65 | 240 | 0.019 |
| IGF1 - S0026 | 157 | 0.12 | 315.09 | 80 | 0.019 |
| IGF1 - S0090 | 157 | 0.52 | 795.66 | 80 | 0.019 |
| IGF1 - S0097 | 157 | nan | 1439.87 | 120 | 0.019 |
| S0005 - S0026 | 157 | 0.06 | 326.62 | 192 | 0.019 |
| S0005 - S0090 | 157 | 0.25 | 567.61 | 192 | 0.019 |
| S0005 - S0097 | 157 | nan | 1348.89 | 288 | 0.019 |
| S0026 - S0090 | 157 | 0.06 | 76.12 | 64 | 0.371 |
| S0026 - S0097 | 157 | 0.23 | 591.15 | 96 | 0.019 |
| S0026 - S0155 | 157 | 0.46 | 1044.58 | 96 | 0.019 |
| S0026 - S0226 | 157 | 0.69 | 1648.39 | 112 | 0.019 |
| S0026 - S0228 | 147 | nan | 2153.84 | 91 | 0.019 |
| S0026 - S0355 | 157 | nan | 2647.7 | 96 | 0.019 |
| S0090 - S0097 | 157 | 0.07 | 291.6 | 96 | 0.019 |
| S0090 - S0155 | 157 | 0.16 | 545.2 | 96 | 0.019 |
| S0090 - S0226 | 157 | 0.42 | 1062.45 | 112 | 0.019 |
| S0090 - S0228 | 147 | nan | 1592.58 | 104 | 0.019 |
| S0090 - S0355 | 157 | nan | 2047.64 | 96 | 0.019 |
| S0090 - Sw122 | 157 | nan | 2626.57 | 96 | 0.019 |
| S0097 - S0155 | 157 | 0.07 | 237.22 | 144 | 0.019 |
| S0097 - S0226 | 157 | 0.16 | 607.88 | 168 | 0.019 |
| S0097 - S0228 | 147 | 0.45 | 1079.1 | 156 | 0.019 |
| S0097 - S0355 | 157 | 0.63 | 1481.8 | 144 | 0.019 |
| S0097 - Sw122 | 157 | nan | 2040.83 | 144 | 0.019 |
| S0155 - S0226 | 157 | 0.07 | 299.82 | 168 | 0.019 |
| S0155 - S0228 | 147 | 0.13 | 566.23 | 156 | 0.019 |
| S0155 - S0355 | 157 | 0.23 | 921.84 | 144 | 0.019 |
| S0155 - Sw122 | 157 | 0.82 | 1495.36 | 144 | 0.019 |
| S0155 - Sw24 | 153 | nan | 2105.85 | 156 | 0.019 |
| S0226 - S0228 | 147 | 0.08 | 290.08 | 182 | 0.019 |
| S0226 - S0355 | 157 | 0.12 | 514.52 | 168 | 0.019 |
| S0226 - Sw122 | 157 | 0.41 | 1028.25 | 168 | 0.019 |
| S0226 - Sw24 | 153 | nan | 1598.06 | 182 | 0.019 |
| S0226 - Sw240 | 157 | nan | 2082 | 182 | 0.019 |
| S0228 - S0355 | 147 | 0.07 | 243.29 | 156 | 0.019 |
| S0228 - Sw122 | 147 | 0.14 | 543.28 | 156 | 0.019 |
| S0228 - Sw24 | 144 | 0.28 | 986.51 | 169 | 0.019 |
| S0228 - Sw240 | 147 | nan | 1456.73 | 169 | 0.019 |
| S0228 - Sw2406 | 147 | nan | 1867.11 | 130 | 0.019 |
| S0355 - Sw122 | 157 | 0.07 | 227.5 | 144 | 0.019 |
| S0355 - Sw24 | 153 | 0.13 | 521.42 | 156 | 0.019 |
| S0355 - Sw240 | 157 | 0.29 | 1027 | 156 | 0.019 |
| S0355 - Sw2406 | 157 | nan | 1444.44 | 120 | 0.019 |
| Sw122 - Sw24 | 153 | 0.08 | 268.02 | 156 | 0.019 |
| Sw122 - Sw240 | 157 | 0.13 | 541.72 | 156 | 0.019 |
| Sw122 - Sw2406 | 157 | 0.33 | 910.15 | 120 | 0.019 |
| Sw122 - Sw2410 | 157 | nan | 1558.72 | 144 | 0.019 |
| Sw122 - Sw632 | 155 | nan | 2170 | 144 | 0.019 |
| Sw24 - Sw240 | 153 | 0.07 | 276.89 | 169 | 0.019 |
| Sw24 - Sw2406 | 153 | 0.14 | 422.85 | 130 | 0.019 |
| Sw24 - Sw2410 | 153 | nan | 1080.17 | 156 | 0.019 |
| Sw24 - Sw632 | 153 | nan | 1614.18 | 156 | 0.019 |
| Sw240 - Sw2406 | 157 | 0.08 | 224.56 | 130 | 0.019 |
| Sw240 - Sw2410 | 157 | 0.16 | 620.07 | 156 | 0.019 |
| Sw240 - Sw632 | 155 | 0.29 | 1071.65 | 156 | 0.019 |
| Sw240 - Sw72 | 157 | nan | 1375.22 | 104 | 0.019 |
| Sw240 - Sw830 | 156 | nan | 2160.85 | 143 | 0.019 |
| Sw2406 - Sw2410 | 157 | 0.08 | 207.34 | 120 | 0.019 |
| Sw2406 - Sw632 | 155 | 0.14 | 565.21 | 120 | 0.019 |
| Sw2406 - Sw72 | 157 | 0.62 | 882.11 | 80 | 0.019 |
| Sw2406 - Sw830 | 156 | nan | 1547.2 | 110 | 0.019 |
| Sw2406 - Sw857 | 157 | nan | 2099.96 | 90 | 0.019 |
| Sw2410 - Sw632 | 155 | 0.07 | 260.32 | 144 | 0.019 |
| Sw2410 - Sw72 | 157 | 0.31 | 454.56 | 96 | 0.019 |
| Sw2410 - Sw830 | 156 | nan | 1052.7 | 132 | 0.019 |
| Sw2410 - Sw857 | 157 | nan | 1569.52 | 108 | 0.019 |
| Sw632 - Sw72 | 155 | 0.07 | 165.73 | 96 | 0.019 |
| Sw632 - Sw830 | 154 | 0.16 | 499.3 | 132 | 0.019 |
| Sw632 - Sw857 | 155 | 0.50 | 1050.98 | 108 | 0.019 |
| Sw632 - Sw936 | 155 | nan | 1659.95 | 144 | 0.019 |
| Sw72 - Sw830 | 156 | 0.07 | 177.96 | 88 | 0.019 |
| Sw72 - Sw857 | 157 | 0.27 | 476.67 | 72 | 0.019 |
| Sw72 - Sw936 | 157 | 0.53 | 1093.7 | 96 | 0.019 |
| Sw72 - Swr1941 | 154 | nan | 1515.68 | 72 | 0.019 |
| Sw830 - Sw857 | 156 | 0.07 | 127.54 | 99 | 0.076 |
| Sw830 - Sw936 | 156 | 0.14 | 621.4 | 132 | 0.019 |
| Sw830 - Swr1941 | 153 | 0.39 | 935.82 | 99 | 0.019 |
| Sw857 - Sw936 | 157 | 0.07 | 176.71 | 108 | 0.019 |
| Sw857 - Swr1941 | 154 | 0.17 | 479.6 | 81 | 0.019 |
| Sw936 - Swr1941 | 154 | 0.07 | 144.35 | 108 | 0.031 |

Not shown are 110 out of 190 pairs wherein an allele at one or both loci is fixed; this test was implemented in LINKDOS (Garnier-Gere and Dillmann, 1992); *p*-values were adjusted using FDR
